# Characterisation of Bespoke Patient-Derived In Vitro Models of Ewing Sarcoma

**DOI:** 10.3390/cancers18030512

**Published:** 2026-02-04

**Authors:** Elizabeth A. Roundhill, Elton J. R. Vasconcelos, John Davies, Susan A. Burchill

**Affiliations:** 1 Children’s Cancer Research Group, Leeds Institute of Medical Research, St. James’s University Hospital, Leeds LS9 7TF, UK; e.a.roundhill@leeds.ac.uk; 2 Leeds Omics, University of Leeds, Leeds LS2 9JT, UK; e.vasconcelos@leeds.ac.uk; 3 Faculty of Biological Sciences, University of Leeds, Leeds LS2 9JT, UK; j.r.davies@leeds.ac.uk

**Keywords:** Ewing sarcoma, patient-derived cultures, next-generation sequencing, in vitro models, preclinical testing, paediatric mesenchymal lineage, single base substitution 5, single base substitution 1, small insertion and deletion signature 1, small insertion and deletion signature 2

## Abstract

**Background/Objectives**: Preclinical models that accurately reflect Ewing sarcoma (ES) will enable the prioritisation of clinically active targeted agents from bench to clinic. To expedite this process, we have established and characterised patient-derived ES cultures (PDES) in vitro. **Methods**: Fluorescence in situ hybridisation, RT-PCR and western blotting were used to examine expression of the pathognomonic EWSR1 fusions. Activation or repression of EWSR1 fusion downstream targets and proliferation was examined by immunofluorescence and immunohistochemistry. Using next-generation sequencing, the DNA and transcriptomic profiles of PDES and cell lines were compared. The response of PDES and cell lines to standard-of-care chemotherapeutics, ionising radiation and investigational drugs was examined. **Results**: All PDES contain EWSR1 fusion DNA, consistent with a diagnosis of ES. EWSR1 fusion gene RNA and protein were detected in 70% and 21% of PDES, respectively. Markers of proliferation and expression of EWSR1 fusion target genes were consistent with the tumours from which PDES were derived (R^2^ = 0.74, *p* < 0.0001) and the paediatric mesenchymal lineage (SBS5 and SBS1, ID1 and ID2). In contrast, the transcriptome of PDES was significantly different from that of cell lines. PDES had a significantly increased doubling time (*p* < 0.00001), decreased expression of Ki67 (*p* < 0.0001) and increased migration (*p* < 0.02) compared to cell lines. Consistent with the longer doubling time, PDES were more resistant to doxorubicin, etoposide and vincristine and ionising radiation (*p* < 0.0001) than cell lines. PDES were sensitive to mTKIs (cabozantinib, lenvatinib, and regorafenib), and trabectedin. The response of PDES to drugs in vitro reflects the clinical experience of patients. **Conclusions**: Models incorporating PDES cells may positively contribute to the preclinical pipeline.

## Simple Summary

Ewing sarcoma is a cancer of young people, for whom the outcome and treatment have not changed significantly in the last 30 years. Furthermore, treatment is frequently associated with life-changing side effects. There is therefore a need for more effective targeted treatments or treatment combinations to improve survival and limit treatment-associated morbidities. A paucity of clinically informative preclinical models has hindered the prioritisation of effective treatments for evaluation in clinical trials. To overcome this problem, we propagated and characterised Ewing sarcoma cells recently isolated from tumours. These cells contain EWSR1 fusions pathognomonic of Ewing sarcoma and share the transcriptome and protein profiles of the tumours from which they were derived. Furthermore, the response of these cells to chemotherapy, ionising radiation and investigational drugs reflects activity reported in patients. These cells are therefore a valuable tool, which could be incorporated into the preclinical pipeline to improve the identification of effective drugs for clinical evaluation.

## 1. Introduction

Ewing sarcoma (ES) is a rare but aggressive cancer presenting in the bone or soft tissue, most common in children and young people aged 10–25 years [1]. Multi-agent systemic chemotherapy, surgery and radiotherapy have improved the outcome for some patients. However, there is heterogeneity in the response of patients to first-line treatment (0–100% tumour necrosis; [2,3,4,5]), and less than 70% of patients will survive 5 years beyond diagnosis. For patients with localised disease, 5 year survival is 60–70% [6]; however, for the 25% of patients who present with metastatic disease only 30–55% will survive [7]. Disease progression occurs in approximately 50% of all patients, usually within two years of diagnosis; only 49% of patients respond to second-line treatment [8]. Overall survival after relapse is 10–15% [8,9], despite multimodality treatment combining chemotherapy, radiotherapy and surgery. Furthermore, systemic treatment can lead to long-term health morbidities, including, nephro- and cardiotoxicities, lower fertility rates, and susceptibility to developing secondary cancers [10,11,12,13,14]. Therefore, there is a need to reduce reliance on current chemotherapy and accelerate the introduction of more effective, targeted treatments to prevent relapse whilst limiting treatment-associated health problems. Despite this clear unmet need, a targeted therapy has yet to become standard of care for patients with ES, reflecting both a limitation of the current pathway to prioritise drugs for evaluation in clinical trials and a lack of an effective targeted treatment. This data illustrates the urgent need for more reliable preclinical models and effective, less toxic treatments.

Development of an ES mouse model has been unsuccessful to date [15]; therefore, preclinical studies of ES have focused on the use of established cell lines both in vitro and in vivo and, more recently, patient-derived xenografts [16]. However, these models are yet to identify a targeted drug that has been significantly effective in clinical trials and changed the standard of care in ES. For example, the PARP inhibitor olaparib and osteoclast inhibitor zoledronic acid both showed promise when evaluated in cell lines in vitro and in vivo cell line xenograft models [17,18]. However, this did not translate to a clinical benefit in ES patients [19,20]. We have hypothesised that failure to predict clinical response using cell lines reflects their clonal nature and adaptation due to long-term culture on plastic, highlighting the need to improve the preclinical testing pathway in ES.

Inconsistencies in the expression profile and behaviour of established cancer cell lines and the corresponding patient tumours are widely accepted. For instance, although cell lines frequently retain driver mutations [21,22], such as the EWSR1-ETS translocation in ES, the long-term culture of cell lines has been associated with the accumulation of additional secondary genomic changes, such as copy number variations and transcriptomic drift [21], when compared to original tumours. These changes in the transcriptome can result in differential regulation of key biological processes such as nucleotide metabolism, oxidative phosphorylation, cell adhesion and communication [23], reducing their value in the preclinical pipeline.

Although cell lines may not faithfully represent tumours, ES patient-derived xenografts (PDX) do replicate the molecular and cellular phenotype of tumours [24]. However, successful engraftment of PDX is observed in only 24% of cases [24]. Furthermore, the maintenance of PDX models is costly and requires specialist facilities [25], making this impractical for most laboratories. Moreover, the loss of sub-clonal heterogeneity, increased chromosomal aberrations (60% of PDX acquire one large chromosomal aberration within a single passage [26]) and replacement of human tumour stromal cells by murine-derived extracellular matrix (ECM) [27] during propagation further limit the value of PDX. Coupled with a moral obligation to minimise the use of mammals, we have sought to develop an in vitro, cost-effective model of ES using cells recently derived from patients. Such cells preserve the stem-like properties of ES [28], maintain cross-talk between cells of the tumour microenvironment and cancer cells [28] and in glioma have been shown to reflect the genotype and transcriptome of tumours [29].

We have previously isolated patient-derived cells from ES that share the EWSR1-ETS DNA fusion with the tumours from which they were derived [30,31] and used them to identify biomarkers of risk and candidate therapeutic targets [30,32]. Despite a more limited replicative capacity compared to cell lines, ES patient-derived cell cultures (PDES) have been employed in drug screening strategies using high-throughput multi-well plates [31,33]. Moreover, PDES, directly isolated from tumours or generated by passage in mice, can be grown in 3D, retaining not only the characteristics of the tumour but also the tumour microenvironment and extracellular matrix proteins. The response of PDES generated from PDX propagated in mice to drugs is also reported to reflect the response of parental PDX [34].

The aim of this study was to establish if PDES represent the tumours from which they were derived and better portray the patient experience following treatment than established cell lines. To do this, PDES and paired tumours were compared for expression of EWSR1 fusion targets and Ki67, a marker of proliferation. In addition to the genome and transcriptome, phenotypes associated with EWSR1 fusion gene expression were investigated in PDES and cell lines. The response of PDES to chemotherapy and ionising radiation used in the treatment of ES and current investigational drugs of interest in the field, was also compared to the patient experience.

In this study we found 79% of PDES do not express the EWSR1-ETS fusion protein, which is associated with heterogeneous expression of downstream targets of the EWSR1 fusion, leading to increased migration (*p* < 0.02) and decreased proliferation (*p* < 0.00001) [35,36,37] compared to cell lines. Proliferation and expression of downstream targets of the EWSR1 fusion in PDES is consistent with the tumours from which the PDES were derived (R^2^ = 0.74, *p* < 0.0001). The observed heterogeneity of expression of the downstream targets of the EWSR1 fusion in PDES and tumours is consistent with the plasticity of ES cells. Importantly, the response of PDES to doxorubicin, etoposide, vincristine, the active metabolite of ifosfamide (4-hyperoxyifosfamide) and zoledronic acid is consistent with reported patient experience. These data demonstrate that PDES are a promising in vitro model that will positively contribute to the preclinical pipeline to reliably identify drugs and drug combinations to improve outcomes for patients.

## 2. Materials and Methods

### 2.1. Cell Culture

The substrate-adherent ES cell lines were cultured as previously described [38]. All ES cell lines contain EWSR1 gene rearrangements and express CD99 in the cell membrane, characteristic of ES [30]. PDES and cell lines are yeast, bacterial, and mycoplasma-free; cultures are tested for mycoplasma every four months using the EZ-PCR mycoplasma test kit (Geneflow, Lichfield, UK). A204 rhabdomyosarcoma cells were used as a positive control for expression of snail family transcriptional repressor 2 (SLUG/SNAI2); MG63 osteosarcoma cells were used as a positive control for zinc finger E-box binding homeobox 2 (ZEB2), lysyl oxidase (LOX), ecto-5′-nucleotidase (CD73) and SRY-box transcription factor 2 (SOX2); A549 adenocarcinoma cells were a positive control for integrin subunit beta 1 (ITGB1); RD-ES cells were used as a positive control for NK2 homeobox 2 (NKx2.2) and were cultured as previously described [38,39]. SH-SY-5Y neuroblastoma cells and HOS osteosarcoma cells were used as negative controls for the EWSR1-FLI1 fusion. SH-SY-5Y [40] and HOS [39] cells were cultured as previously described.

Fresh tumours (*n* = 55) were obtained from patients undergoing surgery between 1993 and 2021 at the Leeds Teaching Hospitals Trust, Royal Orthopaedic Hospital Birmingham and the Freeman Hospital Newcastle [30]. Samples were collected in 15 mL of Leeds Antibiotic Media and transported at room temperature to Leeds, where they were processed immediately [39]. The tumour was divided, and half was mounted in Optimum Cutting Temperature compound (OCT; Merck Biosciences, Aberdeen, UK) and frozen; the rest was placed into tissue culture [29] to generate PDES.

### 2.2. Fluorescence In Situ Hybridisation (FISH) for the EWSR1 Fusion

Cells were incubated with the denatured Vysis EWSR1 Break apart FISH Probe (3N5920, Vysis, Abbott Laboratories Ltd., Sittingbourne, UK) for 16 h at 37 °C as previously described [30]. Cells were mounted in Faramount Mounting Medium (Dako, Agilent Technologies, Cheadle, UK) containing 4′,6-diamidino-2-phenylindole (0.2 μg/mL, Sigma-Aldrich, Dorset, UK) to label nuclei and visualised using the Widefield Fluorescent Inverted Microscope Nikon Eclipse Ti-E (Nikon, Surbiton, UK).

### 2.3. Reverse Transcriptase Polymerase Chain Reaction (RT-PCR) for the EWSR1 Fusion

RNA was extracted using the RNeasy Micro Kit (Qiagen, Manchester, UK), and expression of EWSR1-FLI1/ERG mRNA was confirmed by RT-PCR [30]. Total RNA (50 ng) was reverse transcribed using Superscript^TM^ III Reverse Transcriptase (Life Technologies, Thermo Fisher Scientific, Warrington, UK), and cDNA was amplified using sequence-specific reverse and forward primers and AmpliTaq Gold DNA polymerase (Invitrogen, Thermo Fisher Scientific) [41]. PCR products were separated by 2% agarose (Sigma-Aldrich) gel electrophoresis and visualised, after staining with ethidium bromide (0.5 μg/mL; Sigma-Aldrich), under UV light.

### 2.4. Whole-Genome Sequencing (WGS)

DNA was extracted using the Blood & Cell Culture DNA Mini Kit (Qiagen, Hilden, Germany) from PDES (*n* = 7, CCRG1-L-008, CCRG1-L-014, CCRG1-L-017, CCRG1-L-023, CCRG1-L-066, CCRG1-L-087, CCRG1-L-088) and ES cell lines (*n* = 6). DNA quality checks, library preparation and WGS were carried out by the Novogene Company Limited (Cambridge, UK). Human whole-genome DNA libraries (350 bp) were prepared using the NEBNext library preparation kit (New England Biolabs, Ipswich, MA, USA). The library was quantified with Qubit and RTqPCR; a bioanalyser was used to determine the DNA fragment size and integrity. Quantified libraries were pooled and sequenced on the Illumina High Seq 2000 to generate >210 GB raw data per sample and >700 million reads per sample (mean reads per sample = 769,867,970, range 738,929,435–895,182,217). There was a <0.03% error rate distribution along each read with <0.2% low-quality base reads (N); >99.8% were clean reads.

Read pairs were discarded if either one was contaminated with adapter (>10 nucleotides aligned to the adapter, allowing ≤10% mismatches), if more than 10% of base calls were ambiguous (read as N), or if the proportion of low-quality (Phred quality < 5) bases was over 50% in either read. Filtered reads were mapped to the reference genome (GCh38) using Burrows-Wheeler Aligner (BWA) software [42] to generate BAM files. Subsequently, Sambamba [43] was used to sort BAM files according to chromosome position. GATK [44] HaplotypeCaller was used to call germline SNPs and InDels, while the GATK VariantFiltration module was used to filter germline SNPs and InDels. The filter parameters for SNPs and InDels are shown as follows: SNP: QD < 2.0 || FS > 60.0 || MQ < 40.0 || HaplotypeScore > 13.0 || MappingQualityRankSum < −12.5 || ReadPosRankSum < −8.0 InDel: QD < 2.0 || FS > 200.0 || ReadPosRankSum < −20.0 Control-FREEC [45] and DELLY [46] were used to call germline CNV and SV, respectively. The parameter with window = 2000 and step = 1000 was set in the config file for CNV calling.

The FASTQ files of sequenced PDES and cell lines are available in the Research Data Leeds Repository (University of Leeds), Elizabeth Roundhill, Susan Burchill (2025): Whole-genome sequencing of patient-derived Ewing sarcoma cultures and Ewing sarcoma cell lines is available on request. Software and algorithms used to analyse total RNA sequencing and WGS data are summarised in Table 1.

### 2.5. Genomic Variants and COSMIC Signature Analyses

SNV and InDel variants common to the general population and already annotated in both dbSNP (https://www.ncbi.nlm.nih.gov/snp/; accessed on 5 February 2023) and gnomAD v4.0 (https://gnomad.broadinstitute.org/; accessed on 28 February 2024) were removed. For the former, all “rs” accession numbers were removed from GATK outputs, whereas for the latter, we excluded all variants present in 32% of the adult European population and better represented patients with ES data from participants < 30 years of age. In-house scripts were developed to deal with gnomAD vcf files per chromosome using the following ad hoc filtering strategy syntax: “AF_nfe ≥ 0.02 AND age_hist_het_*n*_smaller + age_hist_hom_*n*_smaller ≥ 91”, where 91 is 2% of the estimated number of Europeans < 30 years within the gnomAD v4.0 dataset. The code used to apply this filtering strategy is provided here (https://github.com/eltonjrv/bioinfo.scripts/blob/master/filter_gt2pct.pl; accessed on 28 February 2024), along with a second script which removes the common variants from our dataset (https://github.com/eltonjrv/bioinfo.scripts/blob/master/remove_gnomAD_vars.pl; accessed on 28 February 2024).

PDES and ES cell lines vcf outputs from the aforementioned scripts were then submitted to MAFtools [47] for an overall summary and classification of variants within coding regions of genes (e.g., missense, nonsense, and frameshift mutations) (https://github.com/eltonjrv/bioinfo.scripts/blob/master/MAFtools.R; accessed on 28 February 2024), as well as to SigProfilerAssignment [54] for assessment of variants across single-base substitutions (SBS), double-base substitutions (SBS), and InDel (ID) COSMIC signatures for human cancer (v3.3) (https://github.com/eltonjrv/bioinfo.scripts/blob/master/SigProfAssig.R; accessed on 28 February 2024). The mutation burden per COSMIC signature was calculated as previously described [48].

### 2.6. Total RNA Sequencing

As previously described [30], total RNA sequencing FASTQ files were aligned to Gencode human 38 release 25 (GCh38_25) by 2-pass alignment using Spliced Transcripts Alignment to a Reference ([55]; STAR).

### 2.7. Differential Expression (DE) Analysis

RNA samples were sequenced in 3 different batches; the combined read count matrix was therefore submitted to batch effect correction using the ComBat_seq function from the SVA Bioconductor package [56], and then batch effect-adjusted counts were used as input for DE analyses, relying on the DeSeq2 negative binomial distribution model through a local fitting type and a 0.01 false discovery rate (FDR) threshold [49]. Normalised read counts were exported as previously described [30].

Read counts were also submitted to a multi-dimensional scaling (MDS) analysis using the plotMDS function from the EdgeR package [50]. Enhanced Volcano (https://bioconductor.org/packages/release/bioc/html/EnhancedVolcano.html; accessed on 28 June 2024) was employed for an overall DE visualisation through volcano plots. Heatmaps were plotted using the pheatmap R package (https://cran.r-project.org/web/packages/pheatmap/index.html; accessed on 28 June 2024), always relying on hierarchical clustering for both rows (genes) and columns (samples).

### 2.8. Gene Set Enrichment (GSE) Analysis

Gene enrichment on differentially expressed gene sets was performed by the clusterProfiler Bioconductor package [53]. We relied on GO, KEGG, and REACTOME functional annotation databases in a single clusterProfiler execution of differentially expressed genes (DEGs), applying gseGO, gseKEGG, and gsePathway functions (https://github.com/eltonjrv/bioinfo.scripts/blob/master/clustProf-gsea.R; accessed on 28 June 2024). Adjusted *p*-values of < 0.01 were considered significant. All R tools described in the previous subsections were run under the R environment version 4.1.

### 2.9. Western Blotting

Western blotting was performed as previously described [30]. Equal protein (25 μg) loading was confirmed by blotting for β-actin (0.4 μg/mL, A5441, Sigma-Aldrich). Membranes were probed for FLI1 (0.1 μg/mL, sc-356, Santa Cruz Biotechnology Inc., Dallas, TX, USA) and ERG (0.5 μg/mL, ab133264, Abcam Plc., Cambridge, UK) overnight at 4 °C. Proteins were detected using goat anti-mouse (1:5000, 170-6516, Bio-Rad, Watford, UK) and goat anti-rabbit (1:5000, 4010-05, Southern Biotech, Homewood, AL, USA) secondary antibodies for 1 h at room temperature. Protein bands were visualised and quantified using the Luminata^TM^ Forte Western HRP Substrate (Millipore, Livingston, UK) and GelDoc Imaging System (Bio-Rad). 

### 2.10. Immunofluorescence

PDES (4 × 10^3^) and ES cell lines (A673, TC-32 and RD-ES; 6 × 10^3^) and positive control cells (A204, A549 and MG63; 8 × 10^3^) were seeded in triplicate into 96-well cellstar^®^ plates (Greiner, Gloucester, UK) and incubated for 72 h. For cell lines, wells were coated with 50 µL foetal calf serum for 1 h prior to seeding. Cells were fixed in 4% paraformaldehyde (in PBS, Sigma Aldrich) for 15 min and permeabilised with 0.1% triton-X (Sigma Aldrich; in PBS) for 10 min at room temperature. For integrin subunit beta 1 (ITGB1), PDES, ES cell lines and A549 positive control cells (2 × 10^4^) were fixed and permeabilised in methanol:acetone (1:1) for 2 × 2 min.

Non-specific secondary antibody binding was blocked by incubation of blots in 10% normal goat serum (S-1000, Vector Laboratories Inc., London, UK) in PBS for 30 min at room temperature. Cells were incubated overnight at 4 °C with primary antibodies for Ki67 (0.46 μg/mL, M7240 clone MIB-1, Dako, Agilent Technologies), CD73 (100 μg/mL, A13821, Antibodies.com), ITGB1 (10.83 μg/mL, ab52971, Abcam Plc.), NKx2.2 (10 μg/mL, ab210463 488-conjugated, Abcam Plc.), LOX (10 μg/mL, ab197061, 647-conjugated, Abcam Plc.), SLUG/SNAI2 (1.75 μg/mL, #9585, C19G7, Cell Signalling Technology, London, UK), SOX2 (0.48 μg/mL, #5049, 488-conjugated, Cell Signalling Technology), ZEB2 (5 μg/mL, PA5-20980, Thermo Fisher Scientific) or corresponding isotype control (Normal Rabbit Serum Ig mix, 086199 (Thermo Fisher Scientific), Negative Control Mouse IgG1, X0931 (Dako), Alexa Fluor 647 and 488 Rabbit IgG isotype controls (ab199093 and ab199091, Abcam Plc.)). Expression was detected for unconjugated primary antibodies using a goat anti-rabbit (4 µg/mL, 488-conjugated, A11034, Alexa Fluor) fluorescent secondary antibody in PBS containing 4′,6-diamidino-2-phenylindole (DAPI; 0.2 µg/mL, nuclear stain, Sigma Aldrich) for 30 min at room temperature.

For dual antibody labelling, PDES were stained for SLUG/SNAI2, detecting the primary antibody using a goat anti-rabbit (4 µg/mL, 647-conjugated, A21245, Alexa Fluor), followed by incubation with the 488-conjugated SOX2 antibody, as described above.

### 2.11. High-Content Imaging (HCI)

Immunofluorescently labelled cells were imaged using the Cell Discoverer 7 High-Content Imaging fluorescent microscope (CD7; Zeiss, Cambridge, UK). Three fields of view were captured per well at 10× magnification using the Zen Blue Imaging Software (version 3.6, Zeiss). Following antibody labelling, the sum of intensity of secondary antibody fluorescence per cell or per nuclei (stained with DAPI) in each field of view was extracted (Zen Blue). All cells incubated with IgG isotype control antibodies were negative for fluorescence. Cell number was counted by calculating the number of DAPI-stained nuclei per field of view. Cells were labelled with TOTO^TM^-3 Iodide (Thermo Fisher Scientific) to identify the cytoplasm of the cell and to determine cell area (Zen Blue).

### 2.12. Doubling Time

PDES (2 × 10^3^) and ES cell lines (5 × 10^3^) were seeded in triplicate into 96-well cellstar^®^ plates and allowed to adhere overnight. Cells were fixed in 4% paraformaldehyde for 15 min, permeabilised in 0.1% triton-X for 5 min and nuclei stained with DAPI for 30 min at room temperature 24 h, 48 h, 72 h, and 96 h after seeding. Cells were counted (DAPI-positive nuclei) at each time point using HCI.

### 2.13. Migration

Migration over 72 h was determined as previously described [39]. Migration index (MI) = total migrated area relative to the size of the spheroid core at 0 h.

### 2.14. Immunohistochemistry

For detection of CD73, ITGB1, Ki67, NKx2.2, LOX, SLUG/SNAI2, SOX2, and ZEB2, tumour sections (5 µm) frozen in OCT were fixed, permeabilised and incubated with primary (unconjugated) and isotype control antibodies as described above. Protein expression was detected using the Mouse or Rabbit EnVision+ System-HRP (DAB) kit (Dako, Agilent, UK) as previously described [30]. Cells were visualised using the Liquid DAB Substrate Chromogen System for peroxidase (Dako), counter-stained with 0.1% Mayer’s haematoxylin at room temperature for 15 s. Cells were visualised by light microscopy (Zeiss Axioplan microscope). The number of positive cells was recorded, scoring 100 cells in 3 independent fields of view.

### 2.15. Treatment of Cells with Drugs and Ionising Radiation

PDES (4 × 10^3^ for drug treatments and ionising radiation for 48 h, and 1 × 10^3^ for ionising radiation for 1 week), TC-32 and RD-ES cell lines (6 × 10^3^ for drug treatments and ionising radiation for 48 h, and 1 × 10^3^ for ionising radiation for 1 week) were seeded in triplicate into 96-well cellstar^®^ plates and allowed to adhere overnight. Cells were treated with doxorubicin (0.00085–10 µM), etoposide (0.007–10 µM), 4-hyperoxyifosfamide (0.16–431 µM), vincristine (0.001–10 µM), the multi-tyrosine kinase inhibitors (mTKIs) carbozantinib, lenvatinib or regorafenib (0.8–50 µM), trabectedin (0.001 nm–1 µM) or zoledronic acid (0.16–10 µM) for 48 h. Cells were treated with ionising radiation (2Gy, 4Gy, and 8Gy) and cultured for 48 h or 1 week. Cells were fixed in 4% paraformaldehyde for 15 min, permeabilised in 0.1% triton-X for 5 min and nuclei stained with DAPI for 30 min at room temperature. Cell number was counted (DAPI positive nuclei) using HCI.

The effect of chemotherapy and ionising radiation on proliferation and expression of activated targets of the EWSR1 fusion was also examined. PDES with detectable expression of EWSR1 fusion activated targets (NKx2.2 and SOX2) and expression of the EWSR1 fusion at the RNA and protein level (CCRG1-L-014), and at the RNA level only (CCRG1-L-017 and CCRG1-L-087) were investigated. It was not possible to establish an EC50 for vincristine and etoposide, therefore CCRG1-L-014, CCRG1-L-017, CCRG1-L-087 were treated with the highest concentration of these drugs achievable in patient plasma (vincristine = 1.48 µM, etoposide = 33.4 µM [57]). Cells were also treated with the EC50 of 4-hyperoxyifosfamide (CCRG1-L-014 EC50 = 105.1 µM, CCRG1-L-017 EC50 = 137 µM, CCRG1-L-087 EC50 = 26.7µM) for 48 h and 8Gy of ionising radiation for 1 week. Expression of Ki67, SOX2, and NKx2.2 in the cells remaining after treatment was determined by immunofluorescence and HCI. The effect of doxorubicin was not investigated due to autofluorescence of the drug.

### 2.16. Statistics

Statistically significant differences were determined using a two-tailed non-parametric Mann–Whitney t-test or analysis of variance (ANOVA) with a Tukey’s post-hoc test. Ki67 positivity in PDES and paired tumours was analysed using a paired t-test. Correlations were determined using a Pearson’s correlation coefficient (r). Non-linear regression analysis was used to calculate EC50s, doubling times (log of exponential growth) and differences in response to chemotherapeutic agents, zoledronic acid, mTKIs, trabectedin and ionising radiation (comparison of curves using the extra sum-of-squares F test). Statistical analyses were performed using GraphPad PRISM 7.03 (GraphPad Software, San Diego, CA, USA).

## 3. Results

### 3.1. PDES Contain an EWSR1 Gene Translocation and Heterogeneously Express EWSR1-ETS RNA and Protein

Thirty-four tumour samples were collected at diagnosis; cells were successfully cultured in 91% (31/34) of cases. Propagation of cells from tumour resections was less successful (33% (6/18)), reflecting the reduced number of viable cells in tumour resections after treatment with chemotherapy. Although the numbers are small, cells were cultured from 100% (3/3) of tumours collected at relapse. PDES were propagated a maximum of 21 times (range 17 to 27) before senescence; cells for the following studies were propagated a minimum of 3 and a maximum of 16 times. Interrogation of WGS data for multiple STR motifs confirmed the PDES and tumours were established from the same person.

**Figure 1:**
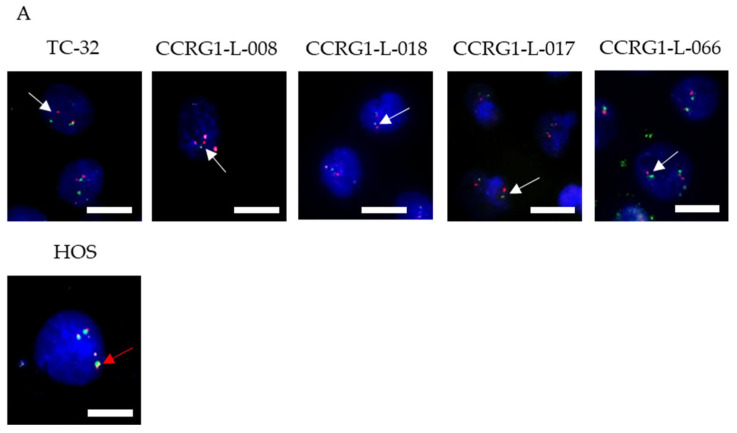

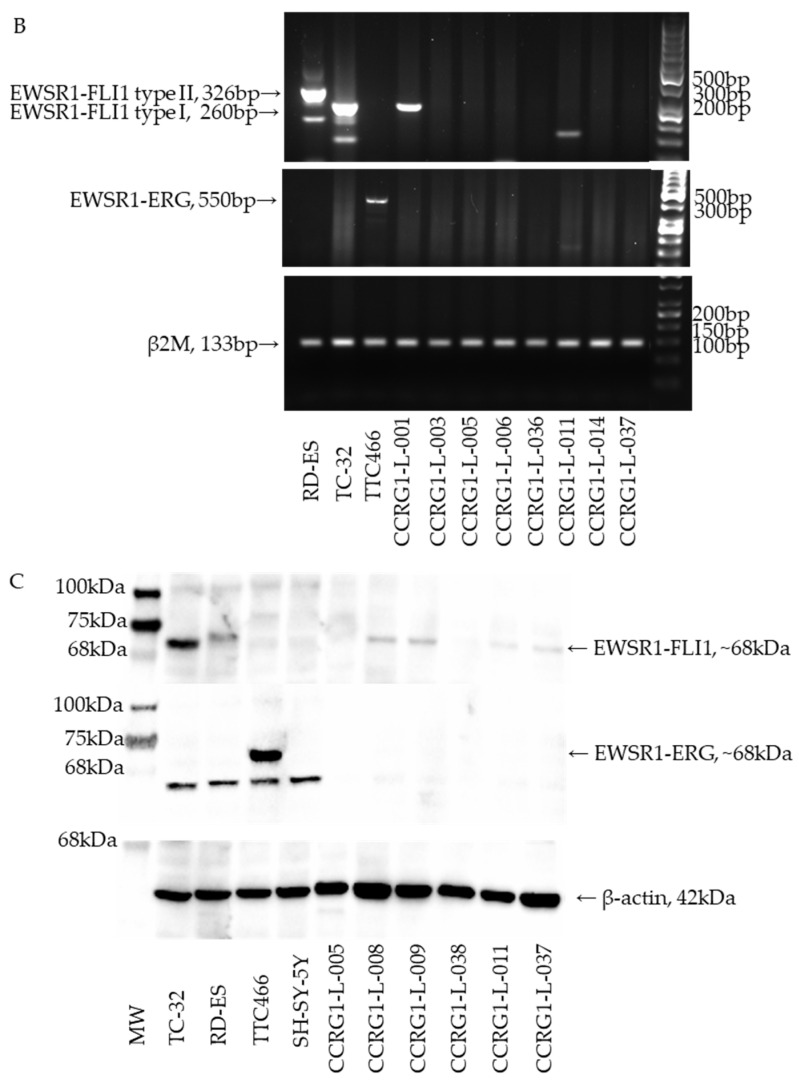
Detection and expression of EWSR1 fusion in PDES. (**A**) Validation of the EWSR1 fusion in PDES using an EWSR1 break-apart probe. PDES = CCRG1-L-008, CCRG1-L-018, CCRG1-L-017, and CCRG1-L-066; TC32 = positive control; and HOS = negative control. White arrows = cells containing both a red and green immunofluorescent signal, indicating an EWSR1 gene translocation. A red arrow = a cell containing a yellow immunofluorescent signal, consistent with an intact EWSR1 probe and no EWSR1 gene translocation. Scale bar = 10 µm. (**B**) EWSR1 fusion RNA detected by RT-PCR after separation of PCR products by agarose gel electrophoresis and staining of DNA with ethidium bromide, visualised under UV light. EWSR1-FLI1 mRNA was detected in 2/8 PDES. TC-32 = EWSR1-FLI1 type I (260 bp) and RD-ES = EWSR1-FLI1 type II (326 bp) positive controls. TTC 466 = EWSR1-ERG-expressing cells (negative control for EWSR1-FLI1). The housekeeping gene B2M was detected in all RNA samples, confirming the presence of RNA/cDNA in the sample and to allow normalisation and quantification of the target transcript. MW = molecular weight markers. (**C**) EWSR1-FLI1 (68 kDa) protein expression in 4/6 PDES detected by Western blot using antibodies to the FLI1 protein. EWSR1-ERG (68 kDa) was not detected in the PDES studied; Western blot using antibodies to ERG. Equal protein loading was confirmed following incubation with an antibody to β-actin. Protein extracts from TC-32 (EWSR1-FLI type 1), RD-ES (EWSR1-FLI type 2) and TTC 466 (EWSR1-ERG) cells were included as positive controls; SH-SY-5Y neuroblastoma cell protein extract was included as a negative control. MW = molecular weight markers. Full Western blot image in Figure S1.

Expression of CD99 (Table 2; [30]) and the presence of an EWSR1 gene fusion was observed in 100% of samples examined (33/33, range 39–100% positive cells per culture; Figure 1A, Table 2, [30]); 25 samples were from tissue collected at diagnosis, 5 from resection and 3 from relapse (Table 2). Evidence of an EWSR1 gene fusion was confirmed in all PDES examined by WGS (*n* = 7), and the breakpoint was consistent with that observed in paired tumours from which they were derived (*n* = 3, Table 2). Cultures containing >70% EWSR1 fusion-positive cells detected by FISH were used in the current study.

EWSR1-FLI1/ERG fusion RNA was detected by RT-PCR in 69% (22/32) of PDES (Figure 1B, Table 2), although on total RNA sequencing only 8% (2/25) of PDES examined contained an EWSR1 fusion. This reflects the difficulty in identifying expression of a gene translocation using NGS technology, where the partner genes are endogenously expressed, and detection of a fusion RNA relies on read coverage across the junction of the two genes. EWSR1-FLI1/ERG protein was detected in 21% (6/29) of PDES (Figure 1C, Table 2). RNA in the absence of protein was observed in 13/29 PDES; the mechanism underlying the lack of protein expression is currently being investigated. In 10/29 PDES, EWSR1-FLI1/ERG fusion was not detected at the RNA or protein level.

In contrast, TC-32 (EWSR1-FLI1 type I), RD-ES (EWSR1-FLI1 type II), and TTC 466 (EWSR1-ERG) ES cell lines, included as positive controls for the most frequently observed ES translocations in patients, expressed both the target fusion RNA and protein. The level of EWSR1 fusion RNA and protein in some PDES was similar to that in cell lines; expression of EWSR1-FLI1 type I RNA in CCRG1-L-001 was similar to that of TC-32 cells (Figure 1C), and levels of EWSR1-FLI1 protein in CCRG1-L-008 and CCRG1-L-009 were similar to those observed in RD-ES (Figure 1C). Fusion gene expression was higher in CCRG1-L-075, isolated from tumour at resection, than the cell lines.

### 3.2. The Proliferation and Expression of EWSR1 Fusion Downstream Targets and the Mutational Signature of PDES Are Characteristic of Tumours

**Figure 2:**
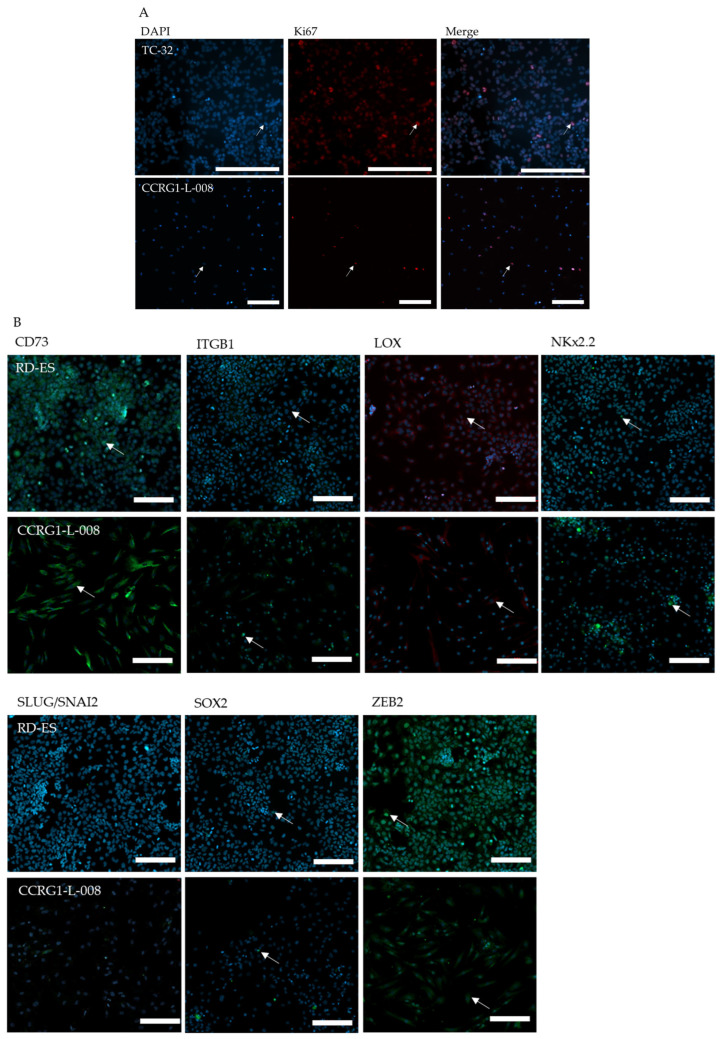

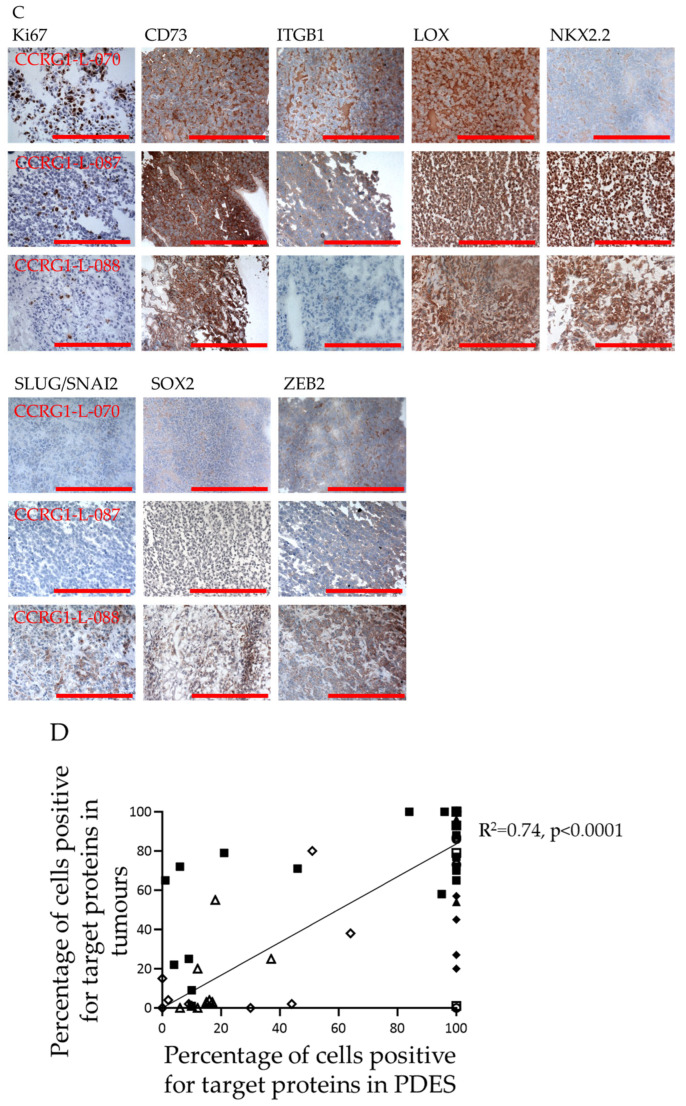

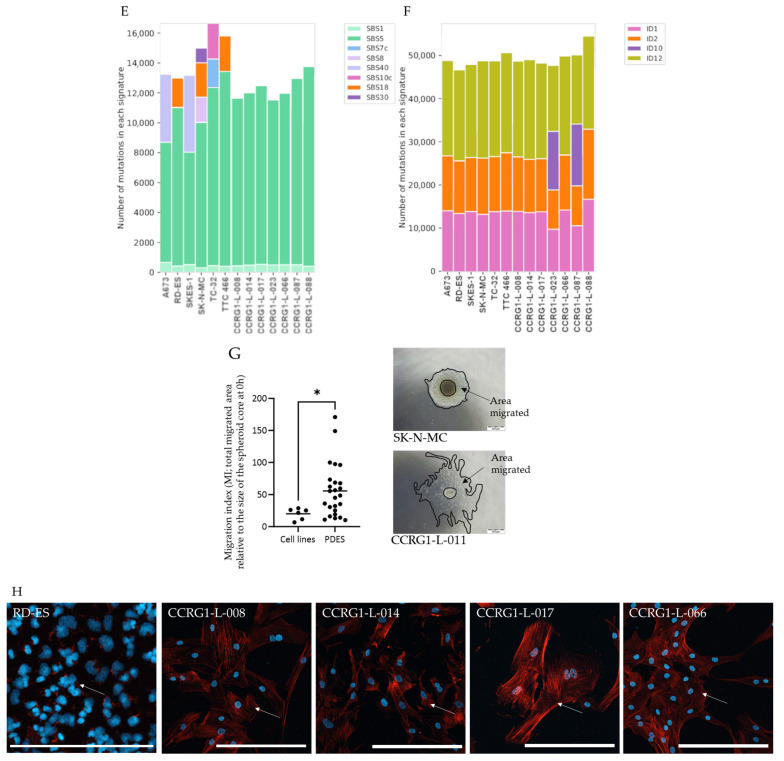
Expression of EWSR1-gene fusion downstream targets, cell proliferation, cell migration and structure of the cytoskeleton in PDES, paired tumours and cell lines. (**A**) Expression of Ki67 in the PDES CCRG1-L-008 and TC-32 cell lines was detected by IF and HCI. Nuclei were stained with DAPI, and three regions were imaged per well across three independent experiments. IgG control cells were negative for fluorescence. White arrows = co-localisation of DAPI and Ki67, white scale bar = 200 µm. (**B**) Protein expression of downstream targets of the EWSR1 fusion (CD73, ITGB1, LOX, NKx2.2, SLUG/SNAI2, and SOX2) and the non-fusion target, ZEB2, in RD-ES cells and CCRG1-L-008 by IF and HCI. White scale bar = 200 µm, white arrows = positive staining in PDES. (**C**) Tumours paired with PDES (CCRG1-L-070, CCRG1-L-087, and CCRG1-L-088) analysed by IHC. Red scale bar = 200 µm. (**D**) There was a positive correlation between the percentage of cells positive for CD73, ITGB1, LOX, NKx2.2, SLUG/SNAI2, SOX2, ZEB2, and Ki67 in PDES (detected by HCI) and paired tumours (detected by IHC); Pearson’s correlation coefficient (R^2^ = 0.70, *p* < 0.0001). Filled triangle = CD73, filled diamond = ITGB1, open triangle = Ki67, open circle = LOX, filled square = NKx2.2, open diamond = SLUG/SNAI2, filled square = SOX2, open square = ZEB2. (**E**) Detection of single base substitutions (SBS) and (**F**) small insertion and deletion (ID) mutational signatures from the COSMIC database in PDES and cell lines. After removal of common variants from WGS data using dbSNP and gnomAD, SBS5, ID1, ID2 and ID12 were the most common signatures in PDES and ES cell lines. ES cell lines contained additional SBS signatures (SBS7c, SBS8, SBS10c, SBS18, SBS30, and SBS40). (**G**) The migration of PDES and cell lines from a 3D spheroid, visualised using light microscopy (Olympus). The migration index (MI) was calculated using image J, expressing the total migrated area relative to the size of the spheroid core at 0 h. Migration was observed in 100% of PDES (25/25) and ES cell lines (6/6). PDES were more migratory than ES cell lines (* = *p* < 0.02). Light microscopy representative images of migrating SK-N-MC (MI = 11.6 ± 1) and PDES CCRG1-L-011 (MI = 149 ± 5) are shown. Black arrow = migration area. (**H**) The actin cytoskeleton of the RD-ES cell line and PDES (CCRG1-L-008, CCRG1-L-014, CCRG1-L-017, and CCRG1-L-066) was determined by IF, staining cells with phalloidin (red) and DAPI (blue). PDES had a more extensive actin cytoskeleton than ES cell lines. White arrow = actin cytoskeleton. The white scale bar = 100 µm.

Consistent with the presence of proliferating cells, expression of Ki67 was detected in 100% (13/13) of PDES examined (range 3–37%, Figure 2A, Table 3). Comparison of Ki67 positivity in 8 PDES (mean percentage of positive cells = 13 ± 2%, Figure 2A, Table 3) and the corresponding tumours from which they were derived (75% (6/8) positive tumours, range 0–55%, mean percentage of positive cells = 10 ± 7%, Figure 2A, Table 4) revealed no significant difference in the percentage of positive cells in PDES and corresponding tumours (*p* = 0.69); the number of proliferating cells in PDES is not statistically different from the number of proliferating cells in tumours. For example, the number of Ki67-positive cells was highest in the paired tumour (55 ± 4%) and PDES CCRG1-L-070 (18 ± 1%) and lowest in the paired tumour (0%) and corresponding PDES CCRG1-L-026 (6 ± 1%; Table 4). These data are consistent with previous publications reporting heterogeneous proliferation in tumours [58,59,60].

Since we observed heterogeneity in RNA and protein expression of the EWSR1 fusion in PDES, the expression of selected downstream targets reported to be activated (NKx2.2 [61], SOX2 [62]) and repressed (CD73 [63], ITGB1 [64], LOX [65], and SLUG/SNAI2 [35]) by the EWSR1 fusion was examined. To further confirm PDES represent the tumours from which they were derived, where possible, levels of the downstream targets of the fusion were also determined in paired tumours by IHC (*n* = 9).

The repressed targets CD73, ITGB1 and LOX were expressed in 100% of PDES and 100% of cells within each PDES population (Figure 2B, Table 4). For activated target NKx2.2 (nuclear), expression was detected in 100% of PDES, and 84–100% of cells per PDES were NKx2.2 positive. Suggesting PDES represent tumours, CD73, ITGB1, and NKx2.2 were also detected in 100% of paired tumours. However, there was heterogeneity in the percentage of cells positive for CD73 (range 53–100%), ITGB1 (range 20–100%), LOX (range 0–100%) and NKx2.2 (range 65–100%) in tumours (Figure 2C, Table 4).

In contrast, the repressed target, SLUG/SNAI2, and activated target, SOX2, were detected in 67% and 100% of PDES and in 0–65% and 1–95% of cells in PDES populations (Figure 2B, Table 4), respectively. Similarly, SLUG/SNAI2 and SOX2 were expressed in 67% and 100% of paired tumours and in 2–80% and 1–79% of tumour cells, respectively (Figure 2C, Table 4). ZEB2, the non-fusion target, was detected in 100% of PDES and paired tumours; however, there was more heterogeneity in the percentage of positive cells (1–100%) in tumours (Figure 2C) compared to the 100% positivity observed in PDES cells (Figure 2B, Table 4).

These data revealed repressed targets of the fusion were highly expressed in 100% of tumours and paired PDES, but the positivity of one of the activated targets, SOX2, was heterogeneous in both datasets. Confirming PDES represent the tumours from which they were derived, the percentage of cells positive for Ki67, the 6 EWSR1 fusion targets and ZEB2 in PDES and paired tumours were correlated (R^2^ = 0.74, *p* < 0.0001; Figure 2D). However, there was heterogeneity in the correlation coefficient across the 9 PDES-tumour pairs examined (R^2^ range 0.49–0.97, *p* = 0.22- < 0.0001; Table 4, Figure S2), suggesting some PDES better represent the original tumour than others. We are currently investigating this heterogeneity using spatial transcriptomics.

After removal of common variants described in dbSNP and gnomAD, the single base substitutions (SBS) in PDES were compared to the 96 signatures in the COSMIC database (with an assignment confidence of >0.85). SBS5, which has no known aetiology, was the dominant signature in PDES (>11,000 mutations; Figure 2E, Table 5). The SBS1 signature was also observed in all PDES but at a lower frequency (<1000 mutations; Figure 2E, Table 5). This is consistent with the known association between SBS1 and the rate of stem cell division and age, reflecting the diagnosis of ES during adolescence. SBS1 is driven by spontaneous or enzymatic deamination of 5-methylcytosine (C) to thymine (T), which is not repaired and persists in the genome. No additional SBS signatures were observed in PDES. In agreement with this data and consistent with a paediatric mesenchymal lineage, SBS1 and SBS5 are the dominant SBS signatures in ES and paediatric solid tumours [66,67,68].

Comparison with the 83 small insertion and deletion (IDs) signatures in the COSMIC database revealed ID1, ID2, and ID12 were the dominant signatures in PDES (assignment confidence of >0.71; Figure 2F, Table 5). ID1 and ID2 signatures have been linked with slippage during DNA replication of the replicated DNA strand and are therefore frequently reported in cancers with DNA mismatch repair deficiency and genomic instability, such as ES [69]. Reflecting our data, ID1 and ID2 signatures are recurrently observed with SBS1 and SBS5 [66]. ID12 was also identified in all PDES (Figure 2F, Table 5), although its aetiology is unknown. Interestingly, the ID10 signature has been identified in solid tumours (COSMIC database), and although the aetiology of ID10 is not known, it was exclusively observed in PDES established from patients with localised tumours (CCRG-L-023 and CCRG-L-087; Figure 2E and Table 5). Doublet base substitution (DBS) signatures were not identified in PDES (Table 5).

### 3.3. The Phenotype of PDES Cells Is Consistent with the Heterogeneous Expression of the EWSR1-Gene Fusion

To investigate if heterogeneous expression of the EWSR1-gene fusion was correlated with cellular phenotype, we examined the growth, proliferation, migration and cytoarchitecture of PDES (*n* = 13) and cell lines (*n* = 6). Consistent with the role of the EWSR1 fusion driving cell growth and proliferation in vitro and in vivo [61,70], PDES (*n* = 13) had an increased doubling time (range 29–234 h; Table 3) compared to ES cell lines (doubling time range 20–33 h; *p* < 0.00001, Table 3). Moreover, expression of the proliferation marker Ki67 was decreased in the PDES (range 3–37%) compared to ES cell lines (range 83–94%; *p* < 0.0001; Figure 2A, Table 3). PDES (*n* = 25, mean MI at 72 h = 56 ± 8, range = 10–171) were more migratory than ES cell lines (mean MI at 72 h = 20 ± 4, range = 7–29, *p* < 0.02; Figure 2G and Table 6), consistent with previous reports that low EWSR1-gene fusion expression is associated with increased migration [35,36,37]. PDES cell area (1801 µm^2^, range 174–3056 µm^2^) was increased compared to that of the cell lines TC-32 and RD-ES (mean area = 249 µm^2^, range 83–675 µm^2^; *p* < 0.0001). Moreover, the PDES had more extensive actin stress fibres, revealed by staining with phalloidin, compared to cell lines (Figure 2H). This observation is consistent with an increase in the actin cytoarchitecture previously reported in conditions of EWSR1 fusion knockdown [35,70].

Interestingly, one PDES (CCRG1-L-075) with higher expression of the EWSR1 fusion compared to other PDES (*p* < 0.05) more closely resembled the established cell lines than the remaining PDES; doubling time (29 ± 6 h), proliferation (37 ± 1% Ki67 positive), and cell size (mean cell area = 369 ± 3 µm^2^). This culture was established from the resection of an ES in the left sacrial iliac of an 11-year-old. The proliferation and migration of cultures CCRG1-L-008 and CCRG1-L-014, which also express the EWSR1 fusion protein, were not significantly different from other PDES. However, the MI was in the range reported in cell lines. This is consistent with the hypothesis that PDES lie in the spectrum between tumours and cell lines. To better understand this relationship and how many PDES are required to model the heterogeneity of ES, we are currently characterising additional PDES-tumour pairs. In summary, PDES express heterogeneous levels of the EWSR1-gene fusion, and in some PDES low levels of the fusion are associated with a low proliferative and highly migratory state [64].

### 3.4. Expression of EWSR1 Fusion Downstream Targets Are Differentially Expressed in PDES Compared to Cell Lines

**Figure 3:**
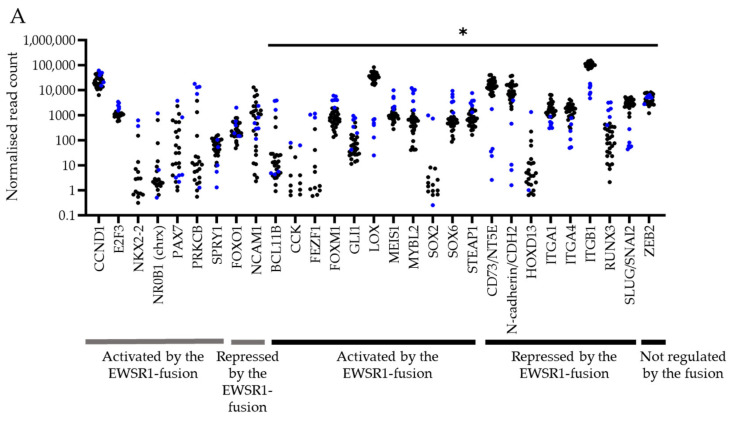

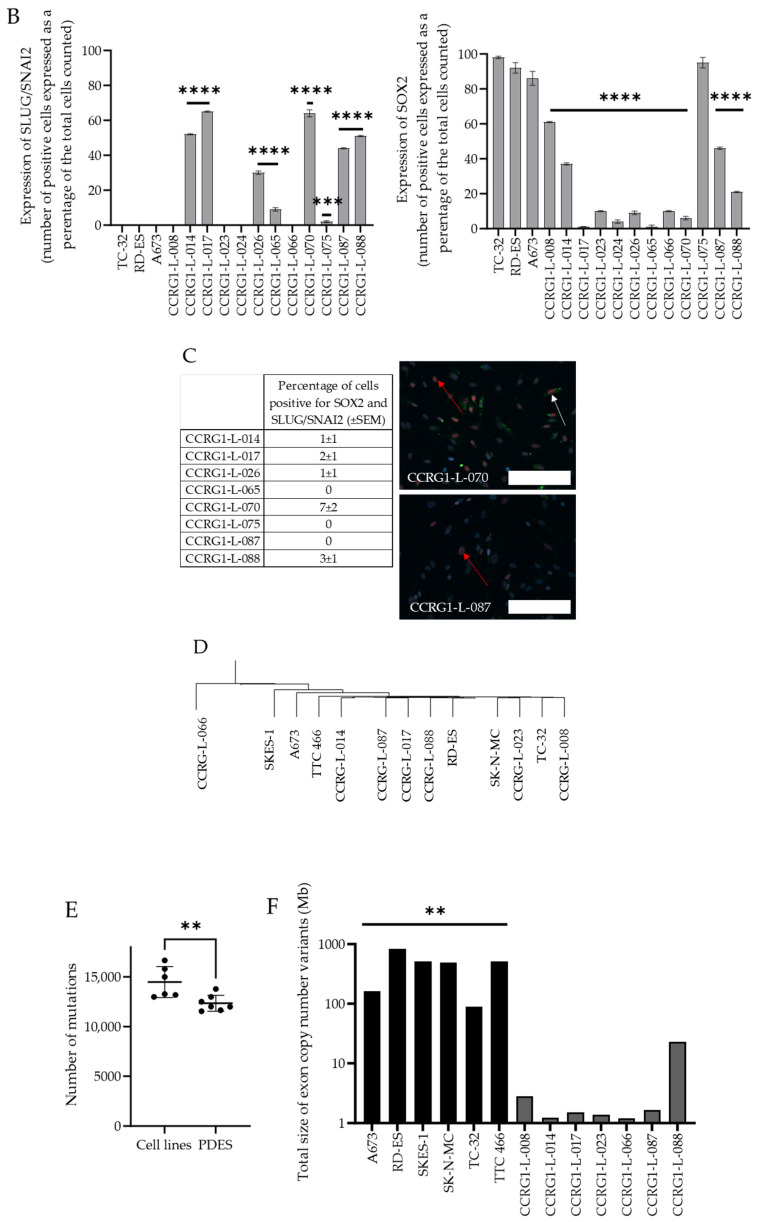
Comparison of downstream targets of the EWSR1 fusion and the genome of PDES and cell lines. (**A**) RNA levels of downstream targets of the EWSR1-FLI1 (as described in Table 2), detected using RNA sequencing and normalised using DESeq2. Levels of 10/17 activated targets were significantly increased, and 9/11 repressed targets were significantly decreased in PDES. Blue circles = ES cell lines, black circle = PDES. Grey line = RNAs shared by PDES and cell lines, black line = RNAs differentially expressed in PDES and cell lines. (**B**) SLUG/SNAI2- and SOX2-positive cells in 12 PDES and A673, TC-32 and RD-ES cell lines, detected by IF and HCI. Results are shown as mean ± SEM, comparing PDES and cell lines. (**C**) The percentage of cells positive for both SOX2 and SLUG/SNAI2 expression (mean ± SEM) in 8 PDES detected using IF and HCI. The proteins were co-expressed in <7% of cells in PDES. Representative images show co-localisation of SOX2 (green) and SLUG/SNAI2 (red) in CCRG1-L-070 cells (white arrow) and in CCRG1-L-070 and CCRG1-L-087 (red arrow) expression of SLUG/SNAI2 only. Scale bar = 200 µm. (**D**) Dendrogram of SNPs from 7 PDES and 6 cell lines detected by WGS. Consistent with a common genetic driver (EWSR1 fusion), PDES and cell lines clustered together. (**E**) Significantly more coding mutations were detected by WGS in cell lines compared to PDES. (**F**) The total size of exon copy number variants in megabases (Mb) was greater in cell lines compared to PDES. * = < 0.05; ** = *p* < 0.01; *** = *p* < 0.001; **** = *p* < 0.0001.

Although the expression of EWSR1 fusion downstream targets in PDES was representative of paired tumours (Table 4), 19/28 EWSR1 fusion target RNAs were differentially activated or repressed in PDES compared to cell lines (adjusted *p* < 0.05, Figure 3A, Table 7). To confirm these findings at the protein level, expression of the targets was compared in cell lines and a wider panel of PDES established from diagnosis tumours that expressed EWSR1 fusion RNA and protein (CCRG1-L-008, CCRG1-L-014, and CCRG1-L-075), at the RNA level only (CCRG1-L-017, CCRG1-L-023, CCRG1-L024, CCRG1-L-066, and CCRG1-L-070) and at neither the RNA or protein level (CCRG1-L-026), in addition to samples from 2 tumours at relapse (CCRG1-L-087 and CCRG1-L-088).

There was no significant difference in the percentage of cells positive or level of expression (sum of fluorescence intensity per cell) of ITGB1, LOX, and NKx2.2 in PDES and cell lines (Table 4 and Figure S3). Furthermore, there was a moderate correlation between expression of SOX2 and Ki67 (R^2^ = 0.69, *p* < 0.0001), although there was no correlation between Ki67 and expression of NKx2.2 (R^2^ = 0.05, *p* = 0.4232). The level of expression of the repressed target CD73 was higher in PDES (*p* < 0.05; Figure 2B and Figure S3) than in cell lines, consistent with the different levels of RNA (Figure 3A).

Consistent with increased expression by the fusion and observed levels of SOX2 RNA (Figure 3A), the percentage of cells positive for SOX2 was higher in ES cell lines compared to PDES (*p* < 0.0001; Figure 2B, Figure 3B and Figure S3). Reflecting RNA levels (Figure 3A), a higher percentage of cells were positive for the repressed target SLUG/SNAI2; 8/12 PDES compared to cell lines (*p* < 0.0001; Figure 2B and Figure 3B), although this was not accompanied by a higher expression level for either protein target (Figure S3).

ZEB2 is not directly regulated by the EWSR1 fusion [37], the percentage of cells positive for this protein (Figure 2B and Figure 3B) and levels of RNA (Figure 3A) were not statistically different in PDES and cell lines. However, the level of ZEB2 protein expression, a transcription factor that represses epithelial gene expression and maintains a mesenchymal state [37], was increased in PDES (*p* < 0.05; Figure S3). This is consistent with the mesenchymal phenotype of PDES cells, which are highly migratory and have an increased actin cytoskeleton and doubling time compared to cell lines, which is likely driven by decreased expression of the EWSR1 fusion [36,63,73].

Similar to observations in PDES and paired tumours, the repressed targets of the fusion CD73, ITGB1 and LOX were also highly expressed in cell lines. However, in contrast, SOX2 was also detected at high levels in established cell lines. Interestingly, >37% SOX2 positivity was detected in CCRG1-L-008, CCRG1-L-014, and CCRG1-L075 (Table 4), PDES where the EWSR1 fusion was detected at the protein level (Table 2). Since SOX2 and SLUG/SNAI2 were the most heterogeneously expressed proteins in PDES and represent both activated and repressed targets of the EWSR1 fusion, respectively, we examined the co-expression of these proteins in PDES. Consistent with opposing transcriptional regulation by the EWSR1 fusion, the proteins were co-expressed in <7% of cells in 8 PDES examined (range 0–7%; Figure 3C).

### 3.5. PDES and Established ES Cell Lines Share SBS1 and SBS5 Mutational Signatures

Next-generation sequencing was employed to profile the genetic and transcriptomic differences giving rise to the contrasting phenotypes observed in PDES and cell lines. Consistent with a shared driver genetic event (EWSR1-ETS fusion), hierarchical clustering of single nucleotide polymorphisms (SNPs) clustered 7 PDES and the 6 ES cell lines together (Figure 3D). However, consistent with the clonal selection of cells following long-term culture, there were significantly more mutations in cell lines (mean number of mutations in all exons = 14,480 ± 1557) than in PDES (mean number of mutations in all exons = 12,351 ± 801, *p* = 0.009, Figure 3E). Furthermore, the size of the exon copy number variants in cell line DNA was significantly larger compared to PDES (*p* < 0.01, Figure 3F). As predicted, mutations in TP53 were observed in 2/6 cell lines (A673, SK-N-MC), STAG2 in 3/6 cell lines (SKES-1, SK-N-MC, and TC-32), and mutations in CDKN2A in 2/6 cell lines (A673 and TC-32). However, most likely reflecting the relative rarity of these mutations in ES [74] and the small sample size in the current WGS study, TP53, STAG2, and CDKN2A mutations were not observed in the 7 PDES subjected to WGS.

After removal of common variants described in dbSNP and gnomAD, the single base substitutions (SBS) in PDES and cell lines were compared to the 96 signatures in the COSMIC database (with an assignment confidence of >0.85). Similar to observations in PDES, SBS5 and SBS1, and ID2 and ID12 were the dominant signatures in cell lines (Figure 2E, Table 5). However, consistent with long-term culture and an increased number and size of mutations, SBS7c, SBS8, SBS40, SBS10c, SBS18, and SBS30 signatures linked with UV- and ROS-induced DNA damage and defective base excision repair were also assigned to the ES cell lines (Table 5). As described above, no additional SBS signatures were observed in PDES.

### 3.6. The Transcriptome of PDES Is Different to That of ES Cell Lines and Enriched for Genes Regulating Cell Migration

The low mutational rate in cancers of young people [75] and our observation that PDES and cell lines share a DNA profile driven by the same pathognomonic translocation and mesenchymal lineage are consistent with the hypothesis that heterogeneity in ES is regulated at the transcriptional level [76,77,78]. Therefore, the transcriptome of 26 PDES and 6 ES cell lines was compared to identify RNAs and corresponding pathways that could be associated with observed differences in cellular morphology and phenotype.

**Figure 4:**
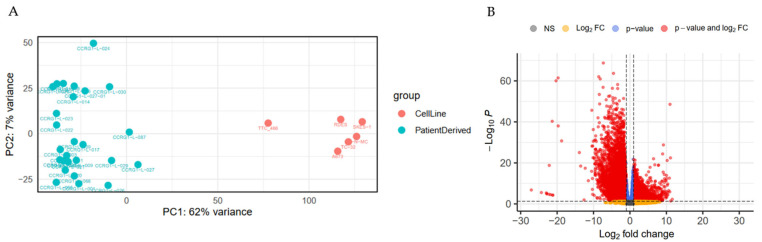

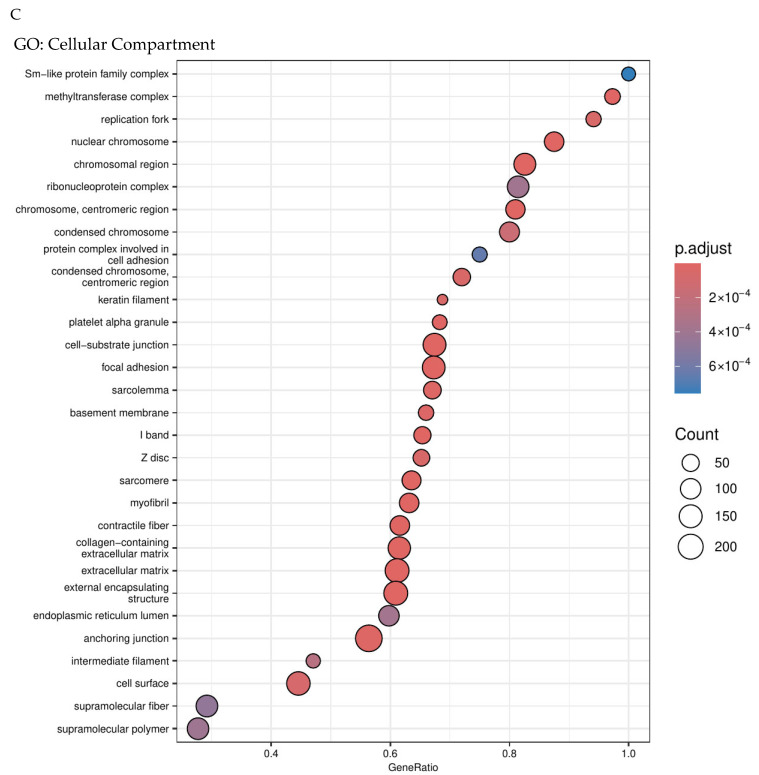

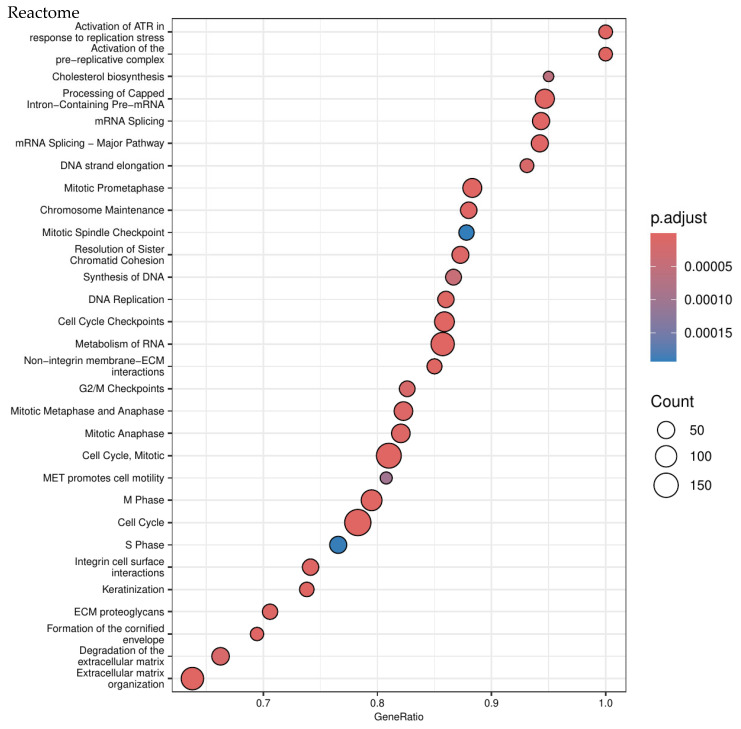
Comparison of the transcriptome of PDES and cell lines. (**A**) Principal component analysis (PCA) of the transcriptome of 26 PDES (turquoise, Table 1) and 6 cell lines (red). PDES clusters independently from cell lines. (**B**) Differentially expressed genes between PDES and cell lines were identified by comparing normalised expression data using DESeq2. There were 15, 121 differentially expressed RNAs with an adjusted *p*-value < 0.01 and log_2_ fold change of >+/−1 (red spots). Blue spots = adjusted *p*-value < 0.01 and a log_2_ fold change of <+/−1; yellow spots = log_2_ fold change of >+/−1 and adjusted *p*-value > 0.01; grey spots = log_2_ fold change of <+/−1 and adjusted *p*-value > 0.01. Positive log_2_ fold change= increased expression in cell lines; negative log_2_ fold change = decreased expression in cell lines. (**C**) Gene set enrichment (GSE) analysis of differentially expressed genes comparing PDES and cell lines was performed using clusterProfiler. Fifty-five percent of pathways identified in gene ontology (GO; Cellular Compartment) and reactome GSE analyses were associated with cell cycle, RNA processing and DNA replication and were upregulated in cell lines compared to PDES. Red to blue = increasing adjusted *p*-value for each pathway; size of bubbles = count of differentially expressed genes in each pathway. Gene ratio = ratio of the number of differentially expressed genes relative to the number of annotated genes in that pathway.

PDES and ES cell lines clustered independently (Figure 4A), reflecting the differential expression of 15, 121 RNAs (*p* < 0.01, log_2_FoldChange > 1 or <−1; Figure 4B, Table S1). Analysis of all DEGs revealed enrichment of pathways associated with increased cell cycle, RNA processing and DNA replication in cell lines compared to PDES (Figure 4C, Table 8 and Table S2), consistent with the decreased doubling time and increased proliferation in cell lines (Figure 2A, Table 3). Supporting the high expression level of the ECM protein, CD73, in PDES compared to cell lines (Figure S3), pathways associated with ECM organisation and structure were also enriched in PDES (Figure 4C, Table 8).

### 3.7. PDES Are More Resistant to Chemotherapy, Ionising Radiation, Zoledronic Acid, mTKIs, and Trabectedin than ES Cell Lines

Since we have shown the COSMIC mutation profile, proliferation and expression of EWSR1 fusion targets in PDES is representative of tumours, we have examined the sensitivity of 8 PDES and 6 established cell lines to cytotoxic chemotherapies and ionising radiation (used as standard of care in ES) and those investigational drugs recently or currently being evaluated in clinical trials.

**Figure 5:**
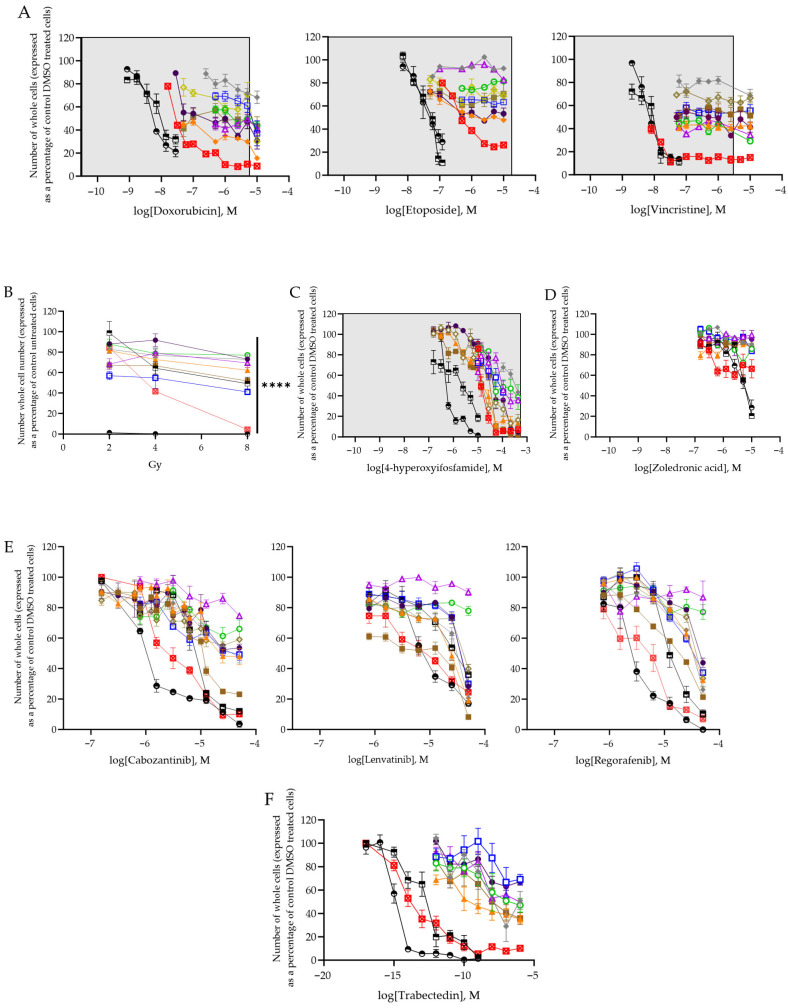

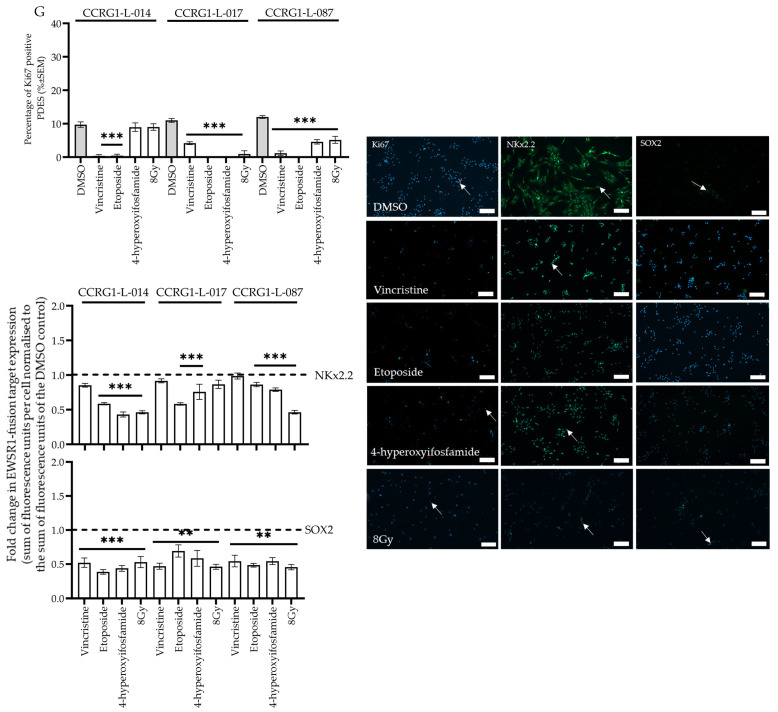
The response of PDES to chemotherapy, ionising radiation, zoledronic acid, multi-tyrosine kinase inhibitors, or trabectedin. Response of PDES, TC-32, and RD-ES cell lines to (**A**) doxorubicin (0.00085–10 µM), etoposide (0.007–10 µM), vincristine (0.001–10 µM), (**B**) irradiation (2–8Gy for 1 week; **** = *p* < 0.0001 compared to TC-32 cells), (**C**) 4-hyperoxyifosfamide (the active metabolite of ifosfamide (0.16–431 µM)), (**D**) zoledronic acid (0.16–10 µM), (**E**) mTKIs cabozantinib, lenvatinib, regorafenib (0.8–50 µM), or (**F**) trabectedin (0.001 nM–1 µM) for 48 h. Cells were labelled with DAPI and TOTO-3 and the number of cells remaining after treatment determined by HCI. Cell number was expressed relative to the vehicle control or non-irradiated cells. PDES (9/9) were more resistant to doxorubicin, etoposide and vincristine than cell lines; 8/9 PDES were more resistant to ionising radiation than ES cell lines. All PDES (9/9) were sensitive to 4-hyperoxyifosfamide but did not respond to zoledronic acid. There was heterogeneity in the response of PDES to mTKIs and trabectedin. Grey shading= where known, concentrations are achievable in-patient plasma. Green open circle = CCRG1-L-008, pink open triangle = CCRG1-L-014, orange filled triangle = CCRG1-L-017, purple filled circle = CCRG1-L-023, brown filled square = CCRG1-L-066, red square with cross = CCRG1-L-075, grey filled diamond = CCRG1-L-087, blue open square = CCRG1-L-088, black half-filled circle = TC-32, black half-filled square = RD-ES. (**G**) Ki67 positive PDES and fold change in fluorescence intensity of the EWSR1 fusion activated targets NKx2.2 and SOX2 in PDES following treatment with vincristine (1.48 µM), etoposide (33.4 µM), or 4-hyperoxyifosfamide (CCRG1-L-014 EC50 = 105.1 µM, CCRG1-L-017 EC50 = 137 µM, CCRG1-L-087 EC50 = 26.7 µM) for 48 h or 8Gy ionising radiation for 1 week (** = *p* < 0.01, *** = *p* < 0.001). The percentage of Ki67-positive cells was decreased in 3/3 PDES following treatment with vincristine or etoposide. Ki67-positive PDES were decreased in CCRG1-L-017 and CCRG1-L-087 after treatment with 4-hyperoxyifosfamide or 8Gy of ionising radiation. Expression of SOX2 was decreased following treatment with all chemotherapeutics or ionising radiation in 3/3 PDES. NKx2.2 expression was decreased in 3/3 PDES following treatment with etoposide or 4-hyperoxyifosfamide, and in 2/3 after treatment with 8Gy of ionising radiation. The fold change in NKx2.2 and SOX2 levels is expressed as the sum of fluorescent units per cell of EWSR1 fusion targets following treatment with chemotherapies or ionising radiation normalised to the sum of fluorescent units per cell of the DMSO or untreated cell control. Representative images of IF for Ki67, NKx2.2 and SOX2 in CCRG1-L-087. Cells were imaged using HCI. Nuclei were stained with DAPI. Three regions were imaged per well, and each experiment was performed in triplicate. IgG control-treated cells were negative for fluorescence. Scale bar = 200 µm, white arrows = positive staining.

PDES express heterogeneous levels of the EWSR1 fusion, and in PDES with low levels, we have shown this results in a more mesenchymal-like, low proliferative and highly migratory state consistent with previous observations [64]. Reflecting the lower proliferation rate, PDES were more resistant to doxorubicin, etoposide and vincristine at concentrations achievable in patient plasma [57] (Figure 5A) than TC-32 and RD-ES cell lines (*p* < 0.0001; Figure 5A, Table 9). So much so, it was not possible to establish an EC50 for doxorubicin, etoposide or vincristine in 8/9 PDES (Table 9), as the whole cell number did not decrease to less than 50%. EC50 values were calculated for CCRG1-L-075, a culture with detectable expression of the EWSR1 fusion protein, the shortest PDES doubling time and the highest Ki67 positivity (Table 3). Cell lines with a higher proliferation and shorter doubling time were more sensitive to doxorubicin, etoposide and vincristine (Table 9).

Eight/9 PDES were more resistant to ionising radiation at 48 h than TC-32 and RD-ES cells (2Gy-8Gy, *p* < 0.0001; Figure S4). Interestingly, only TC-32 cells were significantly more sensitive after 1 week (2Gy-8Gy, *p* < 0.0001; Figure 5B); the percentage of RD-ES cells remaining after 1 week was increased compared to 48 h, suggesting ionising radiation does not abolish the capacity of RD-ES cells to grow. Similar to the response to chemotherapy, CCRG1-L-075 was more sensitive to ionising radiation at all doses and time points compared to other PDES (*p* < 0.0001). However, TC-32 cells were more sensitive than CCRG1-L-075 (*p* < 0.0001, Figure 5B and Figure S4).

The partial resistance to cytotoxic chemotherapy and ionising radiation observed in PDES is more consistent with the heterogeneous patient response to these agents in the clinic [2,3,4,5] than the sensitivity of cell lines. However, in this study there was no association between the response of PDES to chemotherapy or ionising radiation (Figure 5A,B) and tumour necrosis in patients at surgery, their event-free survival or overall survival (Table 9).

Ifosfamide is currently amongst the most promising treatments for patients with relapsed, refractory ES, prolonging patient event-free and overall survival above topotecan/cyclophosphamide in a Phase III comparison [79,80]. Further suggesting the activity of candidate drugs in PDES in vitro may reliably predict clinical activity in patients with ES, PDES were sensitive to 4-hyperoxyifosfamide (the active metabolite of ifosfamide, EC50 range 12.3–105.1 µM; Figure 5C, Table 9). Most likely reflecting the decreased doubling time of cell lines, RD-ES and TC-32 cells were significantly more sensitive to 4-hyperoxyifosfamide than PDES (*p* < 0.0001; Figure 5C, Table 9). Similarly, CCRG1-L-075 was more sensitive to 4-hyperoxyifosfamide than 7/8 PDES (*p* < 0.0001; Figure 5C, Table 9). However, PDES did not respond to zoledronic acid (>60% cells remaining at 10 µM; Figure 5D, Table 9), which is consistent with data from patients in two prospective, multicentre randomised control trials of patients with localised ES (Ewing 2008R1 (EudraCT2008-003658-13)), where the addition of zoledronic acid to maintenance treatment did not improve patient event-free or overall survival [20] but increased renal, neurologic and gastrointestinal toxicities [20]. Consistent with preclinical efficacy using the TC-71 ES cell line [18], zoledronic acid significantly decreased RD-ES and TC-32 cell number (Figure 5D, Table 9). Because zoledronic acid did not decrease PDES cell number by >50%, it was not possible to generate an EC50 or a concentration curve. Therefore, the statistical significance of the response of PDES compared to that of ES cell lines was not compared.

Following encouraging results from single-agent clinical trials where partial tumour responses and an increase in progression free survival was observed [80], the multi-tyrosine kinase inhibitors (mTKIs), cabozantinib (PEMBROCABOSARC NCT05182164, NCT06156410), lenvatinib (rEECur, ISRCTN36453794) and regorafenib (INTER-EWING-1 ISRCTN17938906, NCT05830084, NCT04698785, and NCT04055220), [80] are currently being evaluated in combination with chemotherapies at diagnosis and as a maintenance treatment in ES. Although PDES expressed relatively low vascular endothelial growth factor receptor 1 (VEGFR1), VEGFR2 and VEGFR3 RNA (Table S4), amongst the primary targets of the 3 mTKIs examined, it was possible to generate an EC50 for the same 6/9 PDES following treatment with cabozantinib (2.2–12.2 µM) and lenvatinib (6.4–45.4 µM, Table 9), suggesting these inhibitors have direct effects on the tumour cells or may be binding alternative protein targets (Table S4), highly expressed in PDES. In contrast, an EC50 value was generated in only 2/9 PDES in response to regorafenib, suggesting lenvatinib or cabozantinib might be more useful to treat a wider panel of ES patients. The effect of drugs which target tumour vasculature would be better evaluated in multicellular 3-D spheroids or xenografts. 

Interestingly, EC50 values could not be determined for CCRG1-L-008 and CCRG1-L-014 in response to any of the mTKIs examined. The tumours from which these PDES were derived, were established from two patients that had limited clinical response; tumour necrosis in the CCRG1-L-008 tumour was only 65% (Table 9), patient CCRG1-L-014 had progressive disease, and both patients have died of disease. Moreover, CCRG1-L-014 also had the highest EC50 value for 4-hyperoxyifosfamide, the only chemotherapy that was effective in a concentration-dependent manner in all the PDES studied (Table 9). Of the 9 PDES examined, CCRG1-L-008, CCRG1-L-014, and CCRG1-L-075 all expressed the EWSR1 fusion at the protein level. The sensitivity of CCRG1-L-075 in contrast to the relative resistance of CCRG1-L-008 and CCRG1-L-014 suggests the efficacy of mTKIs may not be linked to expression of the fusion. Consistent with this and previous preclinical in vitro data investigating the efficacy of alternative mTKIs in ES cell lines [81], RD-ES and TC-32 ES cell lines were more significantly sensitive to mTKIs than PDES (Figure 5E, Table 9; *p* < 0.03).

Trabectedin, a drug that decreases expression of the EWSR1 fusion, has shown promise in preclinical studies [82,83]. Moreover, the suppression of genes associated with the DNA damage response by trabectedin makes it an attractive target for combination studies with camptothecins such as irinotecan [82]. This combination has been safely administered to man [84] and is currently being evaluated in a Phase II clinical trial, early results showing a partial response in 5/16 patients ([85]; NCT04067115)). Therefore, we have examined the response of PDES and cell lines to trabectedin. Consistent with previous observations, cell lines were sensitive to trabectedin (TC-32 EC50 = 1 × 10^−7^ µM, RD-ES EC50 = 1 × 10^−8^ µM), as were PDES (Figure 5F, Table 9). CCRG1-L-075 cells with protein expression of the EWSR1 fusion, were more sensitive than PDES with no protein expression (Figure 5F, Table 9; *p* < 0.0001). Similarly, consistent with an increased proliferation rate (Table 4), CCRG1-L-075 was also more sensitive to chemotherapy, ionising radiation, and mTKIs (Table 9) than any other PDES, including those with detectable protein expression of the EWSR1 fusion (CCRG1-L-008, CCRG1-L-014). These observations suggest that the effects of trabectedin are not solely dependent on expression of the EWSR1 fusion proteins.

### 3.8. Proliferating PDES That Express the EWSR1 Fusion Activated Targets, SOX2, and NKx2.2, Are More Sensitive to Chemotherapy and Ionising Radiation

Since cells with increased expression of the EWSR1 fusion are more proliferative [64], we hypothesised that these cells are likely to be more sensitive to treatments targeting highly proliferative cells, such as chemotherapy and ionising radiation.

Consistent with this hypothesis, the percentage of positive Ki67 cells was significantly decreased in all PDES (CCRG1-L-014, CCRG1-L-017, and CCRG1-L-087) after treatment with vincristine and etoposide (*p* < 0.001; Figure 5G) and also in CCRG1-L-017 and CCRG1-L-087 following treatment with 4-hyperoxyifosfamide and ionising radiation (*p* < 0.001; Figure 5G). Moreover, cells remaining after treatment had decreased levels of the EWSR1 fusion activated targets SOX2 (in response to 3/3 chemotherapies and ionising radiation) and NKx2.2 (following treatment with etoposide and 4-hyperoxyifosfamide, and ionising radiation in CCRG1-L-014 and CCRG1-L-08; Figure 5G). These data suggest that cells with low level expression of the EWSR1 fusion, and therefore decreased proliferative capacity [64] are able to persist following treatment with chemotherapy and ionising radiation, suggesting they play a role in disease progression and metastasis.

## 4. Discussion

In this study we confirm that PDES contain the same COSMIC mutational and protein expression signatures as tumours and their response to treatment with cytotoxic chemotherapy and zoledronic acid in vitro reflects the patient clinical experience. Consistent with our previous work using PDES to identify novel biomarkers of risk [30,32], these data suggest PDES could represent a promising preclinical model of ES to prioritise novel drug combinations for evaluation in clinical trials.

Reflecting the presence of the pathognomonic EWSR1-ETS translocation, SBS1, SBS5, ID1, and ID2 COSMIC mutation profiles were the most common signatures observed in PDES and tumours [66,67]). SBS1, SBS5, ID1, and ID2 are the most common signatures in paediatric cancers [66,68] and, consistent with our observations in PDES, are frequently observed together [66]. These mutations are thought to be introduced during DNA replication at mitosis and therefore the signatures are age related, reflecting the number of cell divisions [48]. The ID1 insertion and ID2 deletion of thymine are common to most cancers (except myelodysplastic syndromes and CNS-PiloAstro) accounting for 97% and 45% of InDels in hypermutated and non-hypermutated genomes [48]. Interestingly, PDES cultured from ES tissue at relapse contained more variants than those cultured from tissue collected at diagnosis (Figure S3). Future analysis of paired tumour at diagnosis and relapse will enable a detailed analysis of the mutational profile throughout disease progression.

Reflecting the increased doubling times compared to cell lines, PDES were less responsive to ionising radiation and chemotherapies used in the front-line treatment of ES [86]. Moreover, the PDES remaining after treatment were less proliferative and had lower levels of the EWSR1 fusion target proteins, consistent with the hypothesis that in patients these low-EWSR1 fusion cells survive initial treatment and may be responsible for disease progression and relapse. These data are reflected in the heterogeneity of patient response, assessed by tumour necrosis, to first-line treatment (0–100% tumour necrosis [2,3,4,5]). Since ES cells show evidence of plasticity [87], the transformation of highly migratory, low-EWSR1 fusion persister cells to cells with higher expression of EWSR1 fusion that are less migratory and more proliferative is likely responsible for local or metastatic relapse. Combination treatment to target both the highly proliferative EWSR1 fusion high and low proliferative EWSR1 fusion low cells is therefore a promising strategy to improve outcomes for ES patients.

PDES did not respond to zoledronic acid, and in patients the addition of zoledronic acid to maintenance treatment did not improve patient event-free or overall survival; furthermore, the treatment was associated with increased renal, neurologic and gastrointestinal toxicities [20]. Zoledronic acid inhibits bone resorption, inducing apoptosis of osteoclasts. However, since ES is not derived from osteoclasts and is of mesenchymal origin, it is not unexpected that zoledronic acid has shown limited efficacy in ES. In contrast, PDES were highly sensitive to the active metabolite of ifosfamide (hyperoxyifosfamide) at concentrations achievable in-patient plasma [57]. Consistent with this preclinical activity, ifosfamide has improved survival for patients with ES [88]. More recently, ifosfamide is amongst the most promising treatments for patients with relapsed, refractory ES, prolonging patient event-free and overall survival above topotecan/cyclophosphamide in a Phase III randomised trial [79,80]. In a more targeted approach to treatment, mTKIs are currently being evaluated in combination with chemotherapies at diagnosis and as a maintenance treatment in ES [80]. In 2D culture PDES showed some sensitivity to mTKIs, reflecting previous preclinical activity reported using in vivo xenograft models [89]. Consistent with efficacy in preclinical studies [82,83] and a partial response in patients, ([85]; NCT04067115)), PDES were also sensitive to trabectedin. However, the activity of trabectedin was independent of the basal levels of the EWSR1 fusion protein, consistent with the observation that the activity of trabectedin is not only through inhibition of the EWSR1 fusion [90,91].

Confirming PDES represent the tumours from which they were derived, the expression of downstream targets of the EWSR1 fusion (CD73, ITGB1, LOX, NKx2.2, SLUG/SNAI2, and SOX2), the non-EWSR1 fusion target ZEB2, and the proliferation marker Ki67 in PDES and the tumours from which they were established were correlated. Ki67 positivity in PDES (100% of PDES were positive, across 3–37% of the cells within the tumours) was consistent with the percentage of positive cells previously reported in tumours; 34–100% of tumours were reported to be positive for Ki67 [58,92]), with 0–90% of cells within tumours positive [59,92]. This contrasts with observations in ES cell lines, where 83–94% of cells were positive for Ki67. NKx2.2 was observed in the nucleus of >84% of cells in 100% of PDES and >70% of cells in paired tumours, reflecting its upregulation by the EWSR1 fusion during oncogenesis [87]. Consistent with this, NKx2.2 is frequently described in a high percentage of tumours (90% analysing microarray data [61], 91.2% positive by IHC [93], 93% positive by IHC [94], 93% positive by IHC [95]). It is rarely expressed in other sarcomas or small round cell tumours that are not of mesenchymal origin [61,94] and so is considered a marker of ES in addition to CD99.

However, a second EWSR1 fusion activated target [62,96], SOX2, was heterogeneously expressed in PDES (percentage positive cells per culture ranged from 1–95%) and tumours (percentage positive cells per tumour ranged from 1–79%), consistent with previous observations where 66% [97] and 84.4% of tumours were reported positive for SOX2 by IHC [98]. These data contrast with the expression of the EWSR1 fusion activated NKx2.2, which was identified in a high percentage of PDES and paired tumours. Therefore, consistent with its role in self-renewal and maintenance of embryonic stem cells, it is possible SOX2 is a marker for a subpopulation of cancer stem-like ES cells [97]. This is consistent with an association between high SOX2 expression and tumour relapse (hazard ratio 3.22, *p* < 0.01) [98]. Supporting this hypothesis, in the current study, 2/3 samples from patients at relapse were amongst the most positive for SOX2 expression (CCRG1-L-087 = 71% SOX2 positive, CCRG1-L-088 = 79% SOX2 positive).

Repressed targets of the EWSR1 fusion, CD73, and ITGB1 were expressed in 100% of PDES and paired tumours. Although ITGB1 has not previously been examined in tumours, high levels of CD73 positivity in tumours and PDES (53–100% of cells) were consistent with previous microarray and IHC studies [63] describing inter-tumour heterogeneity. Similarly, the ECM protein LOX was observed in 100% of PDES and 8/9 tumours, consistent with published studies identifying LOX as a target of the EWSR1 fusion [70]. However, RNA levels are reported to be low in ES [65,99], suggesting LOX RNA and protein may not be directly correlated. Consistent with the high expression of the ECM protein CD73 in PDES compared to cell lines (Figure S3), pathways associated with ECM organisation and structure were also higher in PDES compared to cell lines (Figure 4C, Table 8). This association agrees with the importance of the ECM in ES biology.

Protein levels of SLUG/SNAI2 have not previously been examined in tumours. However, in this study consistent with the percentage of positive cells ranging from 0–80% in tumours and PDES, heterogeneous RNA expression has previously been reported in ES cell lines and at decreased levels compared to ZEB2 [37]. ZEB2 is a transcription factor responsible for repressing the epithelial phenotype of cells, and so consistent with the mesenchymal origin of the ES, ZEB2 was detected in 100% of PDES and tumours. This is in keeping with previous observations at the RNA level in tumours and the protein level in cell lines (A673, TC71 and SK-N-MC) where expression was detected in 100% of samples [37].

In addition to confirming PDES represent tumours, protein expression profiles of downstream targets of the EWSR1 fusion were compared in PDES and cell lines, revealing PDES have a more mesenchymal-like phenotype. Specifically, the percentage of SOX2-positive cells was decreased in 11/12 PDES, and the repressed target CD73 was increased in all PDES examined compared to cell lines. Moreover, the number of cells positive for the repressed target SLUG/SNAI2 was also significantly increased in 8/12 PDES compared to cell lines. Further suggesting PDES represents a more mesenchymal-like population, levels of the epithelial phenotype repressor ZEB2 [37] were increased in PDES. Supporting this data and previous observations in low-EWSR1 fusion cells, PDES have an increased cell area and doubling time, decreased proliferation (Ki67 positivity), were more migratory and had a more robust actin cytoskeleton than cell lines.

The expression of EWSR1 fusion downstream targets and the phenotype of PDES are consistent with previous data in cell lines with decreased expression of the EWSR1 fusion. Knockdown of EWSR1-FLI1 [35,37,70] or downstream targets of the fusion (SOX2 [96], NKx2.2 [36]) in ES cell lines decreased cell proliferation [96] and increased anchorage-independent growth, cell area, migration, the number of focal adhesions, and the metastatic phenotype of cells [35,36,37,70]. Similarly, expression of EWSR1-FLI1 in mesenchymal stem cells (MSCs) induced expression of the fusion-activated targets NKx2.2 and SOX2 and decreased cell size, inducing a rounded morphology [100]. Interestingly, high levels of LOX, such as those we have observed in PDES, have been linked with increased migration, invasion and metastasis in ES and the LOX protein is frequently colocalised with actin stress fibres [70].

Recently, CD73 has been identified as a marker of ES cells with increased mesenchymal-like characteristics, a more complex cytoskeleton and high expression of EWSR1 fusion repressed targets and ECM proteins [63]. In previous studies, CD73 expression in tumours was heterogeneous, and levels of cell surface CD73 were low in ES cell lines [63]. These observations are consistent with our findings in tumours, PDES and the predominantly intracellular CD73 expression in cell lines. Furthermore, consistent with the expression profile of PDES in this and our previous studies [30], genes associated with the ECM, LOX, FBN1 and COL6A1, have also been observed at high levels in CD73 positive ES cells [63].

Integrin family members, such as ITGB1, an essential component of the ECM, are also upregulated in EWSR1 fusion low cells [64]. ZEB2 is highly expressed in bone marrow MSCs and neural crest stem cells [37] and in ES by repression of epithelial gene expression [37], balancing the block of MSC features via the EWSR1 fusion and NKx2.2 [87], maintaining ES cells in a partially undifferentiated state [36]. This phenotype is consistent with our observations in PDES, where low expression of the EWSR1 fusion correlates with increased expression of ZEB2. However, we did not observe a difference in NKx2.2 expression.

Reported repressed targets of the fusion were highly expressed in tumours, paired PDES and cell lines, although there was greater heterogeneity in the expression of the activated targets. This reflects the limitations of the strategy used to identify EWSR1 fusion downstream targets, performing knockdown studies in established ES cell lines that do not wholly represent the expression of the targets in tumours. Our observations are consistent with recent reports describing heterogeneous expression of NKx2.2 and the additional EWSR1 fusion target, NR0B1; 20–80% (NKx2.2) and 0–60% (NR0B1) of tumour cells were positive [60]. Taken together, the phenotype and differential expression of EWSR1 fusion downstream targets in some PDES are consistent with a more mesenchymal-like and highly migratory cell state. Since metastasis represents a major challenge for a cure in ES, these PDES represent an excellent model for the preclinical testing of novel agents to target and prevent the dissemination of disease and relapse.

Despite expression of the EWSR1 fusion at the protein level in 25% (2/8) of PDES examined, the transcriptome, phenotype and drug response of the two cultures CCRG1-L-008 and CCRG1-L-014 was different to those observed in cell lines. These PDES appear to lack components that drive ES cells towards a highly proliferative and decreased migratory state. However, for some PDES, such as CCRG1-L-075, the proliferation and expression of SOX2 (EWSR1 fusion activated target) resembled that reported in cell lines. Therefore, the methodology used to isolate PDES from tumours is not selecting for low EWSR1 fusion cell populations. Rather, we suggest that the heterogeneity of EWSR1 fusion expression observed in PDES reflects that observed in tumours, with ES cells regulating the expression of the EWSR1 fusion as required during the multi-step process of cancer evolution and interactions with the tumour microenvironment.

The long-term culture of cell lines has been associated with the accumulation of additional secondary genomic changes, such as copy number variations and transcriptomic drifts [21], compared to original tumours. This is consistent with our observation that cell lines contain more complex mutations than PDES and changes at the transcriptome level that result in differential regulation of key biological processes, including nucleotide metabolism, oxidative phosphorylation, cell adhesion and communication [23]. Importantly in the current study, GSE analysis revealed these pathways were differentially expressed in cell lines compared to PDES, cell lines cultivating a permissive cellular environment for the development of a highly proliferative phenotype. Although PDES better represent tumours than cell lines, a limitation of all preclinical models is that it is only possible to study those cells that survive in the in vitro or in vivo culture conditions. Moreover, although we have collected tumour samples from all comers with ES in the UK, understanding of the intra-tumoural heterogeneity is limited by the single tumour sample received from each patient. Expanding the number of samples collected per tumour will increase understanding of the intra-tumoural heterogeneity.

Some PDES populations contained a low percentage of cells with the EWSR1 fusion DNA, consistent with a higher proportion of TME cells. In this study we have examined PDES with a high percentage of ES cells (>70% EWSR1 fusion DNA positive) with <30% of TME cells. The incorporation of cells of the TME into preclinical models will improve the development of a more representative model. To do this, profiling of all PDES cell populations and paired tumours using WGS, single-cell spatial transcriptomics [60,101] and methylomics will be important. This strategy will allow the construction of bespoke preclinical models, containing PDES and TME cells in a ratio that represents the original cell populations in the paired tumours. In addition, all PDES form 3D spheroids, which more closely represent tumours than cells in 2D and can be cultured on scaffolds [102], allowing us to advance the model by mimicking the cell–cell interactions and biophysical support of the bone. We are currently investigating these possibilities.

Monitoring the transcriptome and methylome of PDES over time will allow the detection of transcriptional and/or post-transcriptional changes introduced as a consequence of propagation in in vitro culture, allowing us to study the evolution of PDES and compare this to established cell lines [21]. As the value of the PDES depends on how faithfully they represent the tumours from which they arise, it is important to establish whether and for how long the PDES retain their genomic, epigenetic and phenotypic characteristics following propagation. We are therefore comparing early and late passages of PDES using single-cell transcriptomics. These studies will also allow the characterisation of the different cell populations in the PDES and establish which, if any, cells are lost or enriched in vitro. We will also compare the stability and fidelity of PDES to that of ES PDX in vivo [24], which are limited by low engraftment rates [24], high costs [25], rapid loss of subclonal heterogeneity, and increased chromosomal aberrations [26], in addition to the replacement of human tumour stromal cells with murine-derived ECM [27]. Our studies suggest that clinically annotated and genomically characterised PDES represent a promising preclinical tool to study ES biology, identify new drug targets and identify existing drugs that eradicate ES cells by exploiting tumour vulnerabilities and drug-repositioning strategies.

## 5. Conclusions

In established PDES we have demonstrated that the proliferation and expression of downstream targets of the EWSR1 fusion are consistent with the tumours from which the PDES were established. Furthermore, the response of PDES to the standard of care treatments and investigational drugs is consistent with the patient experience. The SBS and ID COSMIC mutational profile of PDES is also consistent with a paediatric mesenchymal lineage. The heterogeneity of EWSR1 fusion-activated targets in PDES and tumours is consistent with the hypothesis that ES cells are in a state of plasticity, capable of up- and down-regulating expression of the EWSR1 fusion protein. Moreover, the survival of low-EWSR1 fusion-expressing cells, with decreased proliferation following treatment with chemotherapy and ionising radiation, is consistent with the suggestion that, in patients, cells with these characteristics may persist following treatment and could be responsible for disease progression and relapse. High levels of proliferation and expression of SOX2 and SLUG/SNAI2 in established cell lines do not reflect tumours or PDES. Moreover, cell lines have accumulated additional SBS and ID COSMIC signatures that are not observed in paediatric tumours, suggesting the cell lines do not faithfully represent ES. Using PDES, we have previously identified and validated biomarkers of risk and candidate therapeutic targets [30,32]. Taken together, these data suggest PDES are a promising in vitro preclinical model that, in 2D or 3D, will contribute to the prioritisation of treatments for evaluation in clinical trials.

## Figures and Tables

**Table 1:** Software and algorithms used to analyse total RNA sequencing and whole-genome sequencing data.

Software and Algorithms		
BWA	[42]	https://github.com/lh3/bwa (accessed 5 February 2023)
GATK	[44]	https://gatk.broadinstitute.org/hc/en-us (accessed on 5 February 2023)
FREEC	[45]	https://boevalab.inf.ethz.ch/FREEC/ (accessed on 5 February 2023)
DELLY	[46]	https://github.com/dellytools/delly (accessed on 5 February 2023)
MAFtools	[47]	https://bioconductor.org/packages/release/bioc/html/maftools.html (accessed on 28 February 2024 )
SigProfilerAssignmentR	[48]	https://github.com/AlexandrovLab/SigProfilerAssignmentR (accessed on 28 February 2024 )
DESeq2	[49]	https://bioconductor.org/packages/release/bioc/html/DESeq2.html (accessed on 28 February 2024 )
EdgeR	[50]	https://bioconductor.org/packages/release/bioc/html/edgeR.html (accessed on 28 June 2024)
EnhancedVolcano	[51]	https://bioconductor.org/packages/release/bioc/html/EnhancedVolcano.html (accessed on 28 June 2024)
Pheatmap	[52]	https://github.com/raivokolde/pheatmap (accessed on 28 June 2024)
clusterProfiler	[53]	https://bioconductor.org/packages/release/bioc/html/clusterProfiler.html (accessed on 28 June 2024)

**Table 2:** Summary of EWSR1 fusion detection (DNA) and expression at the mRNA and protein level in 33 PDES and 6 ES cell lines. The presence of an EWSR1 fusion at the DNA level was examined by fluorescence in situ hybridisation (FISH) and whole-genome sequencing (WGS). RNA expression of the EWSR1 fusion was examined by RT-PCR and total RNA sequencing. Protein expression of the EWSR1 fusion was examined by Western blot (WB), and expression of CD99 by immunocytology (ICC). The timepoint tumours were collected to generate PDES is shown. NA = PDES not analysed. PDES were passaged a minimum of 3 and a maximum of 16 times; see Table S1.

Patient-Derived Sample Name	Sample Timepoint (1 = Diagnosis, 2 = Resection, 3 = Relapse)	EWSR1 Status						CD99 Protein Expression
		DNA			RNA		Protein	
		FISH (1= Cells Positive for *EWSR1* Breakapart Probe, 0 = Negative)	Percentage of Cells per Culture with Evidence of an *EWSR1* Breakapart Probe	Whole-Genome Sequencing (1 = EWSR1 Fusion-Positive and Identical Breakpoint Identified in Paired Tumour (Breakpoint Location Described), 2 = EWSR1 Fusion-Positive but Paired Tumour Not Available for Analysis, 3 = Cell Line, EWSR1 Fusion Transcript Type Consistent with Literature)	RT-PCR (0 = Negative, 1 = Positive EWSR1-FLI1 Type I, 2 = EWSR1-FLI1 Type II, 3 = EWSR1-ERG, 4 = Other)	Total RNA Sequencing for EWSR1 Fusion (1 = Positive, 0 = Negative)	WB (0 = Negative, 1 = EWSR1-FLI1 Type 1 or Type 2, 2 = EWSR1-ERG, ND = Not Done)	ICC; Intensity of Staining; 0 = Negative, 1 = Low Expression, 2 = Medium Expression, 3 = High Expression
A673	1	1	100	3 (EWSR1-FLI1 type I)	1	1	1	3
RD-ES	1	1	100	3 (EWSR1-FLI1 type II)	2	1	1	3
SKES-1	1	1	100	3 (EWSR1-FLI1 type II)	2	1	1	3
SK-N-MC	1	1	100	3 (EWSR1-FLI1 type I)	1	1	1	3
TC-32	1	1	100	3 (EWSR1-FLI1 type I)	1	1	1	3
TTC 466	1	1	100	3 (EWSR1-ERG)	3	1	2	3
CCRG1-L-001	1	1	100	NA	1	0	NA	1
CCRG1-L-005	3	1	95	NA	0	0	0	1
CCRG1-L-006	1	1	100	NA	0	0	NA	1
CCRG1-L-008	1	1	91	2	1	0	1	1
CCRG1-L-009	2	1	94	NA	1	0	1	1
CCRG1-L-011	1	1	94	NA	1	0	1	1
CCRG1-L-014	1	1	91	2	1	0	1	1
CCRG1-L-016	1	1	76	NA	1	0	0	2
CCRG1-L-017	1	1	71	2	1	0	0	1
CCRG1-L-018	1	1	70	NA	0	1, CAMSAP2--EWSR1, ETV5--EWSR1	0	2
CCRG1-L-019	1	1	81	NA	0	1, EWSR1--CHD4	0	2
CCRG1-L-020	1	1	72	NA	1	0	0	2
CCRG1-L-021	1	1	85	NA	1	0	0	2
CCRG1-L-022	1	1	94	NA	1	0	0	2
CCRG1-L-023	1	1	93	1 (EWSR1 intron 8/9, FLI1 exon 9)	2	1, SLC6A6--EWSR1	0	1
CCRG1-L-024	1	1	80	NA	2	1, EWSR1-FLI1, TEAD1--EWSR1	0	3
CCRG1-L-025	1	1	92	NA	0	0	0	3
CCRG1-L-026	1	1	87	NA	0	0	0	2
CCRG1-L-027	1	1	85	NA	0	0	0	2
CCRG1-L-028	1	1	84	NA	0	0	0	3
CCRG1-L-029	1	1	100	NA	0	?	0	2
CCRG1-L-030	1	1	100	NA	2	0	0	3
CCRG1-L-039	1	1	91	NA	1	1, SF3B3--EWSR1	1	1
CCRG1-L-064	2	1	99	NA	3	NA	0	3
CCRG1-L-065	2	1	79	NA	NA	NA	NA	1
CCRG1-L-066	1	1	77	2	3	NA	0	2
CCRG1-L-067	1	1	39	NA	3	NA	0	3
CCRG1-L-070	1	1	80	NA	2	NA	0	3
CCRG1-L-071	1	1	93	NA	2	NA	NA	3
CCRG1-L-072	2	1	52	NA	0	NA	0	3
CCRG1-L-075	2	1	100	NA	1	NA	1	3
CCRG1-L-087	3	1	78	1 (EWSR1 exon 7, FLI1 exon 5 (EWSR1-FLI1 type II))	2	NA	0	3
CCRG1-L-088	3	1	83	1 (EWSR1 intron 9/10, NFATC2 intron 3/4)	4	NA	0	3

**Table 3:** Doubling time and proliferation of PDES and cell lines. To calculate doubling times, 13 PDES and 3 ES cell lines (TC-32, RD-ES, and A673) were fixed 24 h, 48 h, 72 h, and 96 h after seeding, nuclei stained with DAPI, and the number of cells counted using HCI (CD7). To identify proliferating cells, cells were fixed, nuclei stained with DAPI and Ki67 (0.46 μg/mL, M7240), and positive cells counted using HCI. ES cell lines had a shorter doubling time and increased expression of Ki67 compared to PDES; results are shown as mean percent ± SEM. Three regions were imaged per well, across three wells. Cells incubated with the IgG control were negative for fluorescence. **** = *p* < 0.0001 comparing the log of exponential growth of PDES and cell lines. ns = not significant.

Cell Culture	Ki67 Positive Cells (Mean % ± SEM)	Doubling Time (h)
TC-32	88 ± 1 ****	33 ± 2 ****
RD-ES	94 ± 1 ****	33 ± 3 ****
A673	83 ± 6 ****	20 ± 2 ****
CCRG1-L-005	3 ± 1	195 ± 21
CCRG1-L-008	20 ± 1	234 ± 40
CCRG1-L-014	14 ± 1	207 ± 30
CCRG1-L-017	11 ± 1	62 ± 9
CCRG1-L-023	15 ± 1	81 ± 28
CCRG1-L-024	10 ± 1	194 ± 13
CCRG1-L-026	6 ± 1	140 ± 21
CCRG1-L-065	12 ± 1	180 ± 18
CCRG1-L-066	17 ± 2	70 ± 13
CCRG1-L-070	18 ± 1	118 ± 5
CCRG1-L-075	37 ± 1	29 ± 6 (ns)
CCRG1-L-087	12 ± 2	145 ± 24
CCRG1-L-088	16 ± 1	112 ± 13

**Table 4:** Expression of Ki67 and EWSR1 fusion target proteins in cell lines, PDES and paired tumours. The percentage of cells positive for the EWSR1 fusion activated and repressed targets (CD73, ITGB1, LOX, NKx2.2, SLUG/SNAI2, and SOX2), the non-fusion target ZEB2 and the proliferation marker Ki67 in 3 ES cell lines, 13 PDES and 9 PDES-tumour pairs were detected by IF and IHC. The correlation between protein expression in PDES and paired tumours was examined by Pearson’s correlation coefficient; R^2^ and corresponding *p*-values for each PDES-tumour pair are shown. There was a significantly positive correlation between 5/8 PDES and paired tumours (CCRG1-L-023, CCRG1-L-026, CCRG1-L-066, CCRG1-L-075, and CCRG1-L-087). The expression of the EWSR1 fusion at the RNA (RT-PCR) and protein (WB) level is shown. 1 = expressed, 0 = no expression. **** = *p* < 0.0001.

	Ki67 (Nucleus)		CD73 (Plasma Membrane, Nucleus, Cytoplasm)		ITGB1 (Plasma Membrane and Cytoplasm)		LOX (Cytoplasm)		NKx2.2 (Nucleus and Cytoplasm)		SLUG/SNAI2 (Nucleus)		SOX2 (Nucleus)		ZEB2 (Nucleus and Cytoplasm)		EWSR1 Fusion Status		R^2^ Correlation Coefficient Comparing PDES and Corresponding Tumours	*p*-Value
Cell Culture	PDES	ES Tissue	PDES	ES Tissue	PDES	ES Tissue	PDES	ES Tissue	PDES	ES Tissue	PDES	ES Tissue	PDES	ES Tissue	PDES	ES Tissue	RNA Detected by RT-PCR (1 = Yes, 0 = No)	Protein Detected by WB (1 = Yes, 0 = No)		
TC-32	88 ± 1 ****	NA	100 ± 0	NA	100 ± 0	NA	100 ± 0	NA	100 ± 0	NA	0	NA	98 ± 2	NA	97 ± 5	NA	1	1	NA	NA
RD-ES	94 ± 1	NA	100 ± 0	NA	100 ± 0	NA	100 ± 0	NA	100 ± 0	NA	0	NA	92 ± 3	NA	100 ± 0	NA	1	1	NA	NA
A673	83 ± 6	NA	92 ± 3	NA	100 ± 0	NA	100 ± 0	NA	100 ± 0	NA	0	NA	86 ± 4	NA	92 ± 3	NA	1	1	NA	NA
CCRG1-L-005	3 ± 1	NA	NE	NA	NE	NA	NE	NA	NE	NA	NE	NA	NE	NA	NE	NA	NE	NE	NA	NA
CCRG1-L-008	20 ± 1	NA	100 ± 0	NA	100 ± 0	NA	100 ± 0	NA	100 ± 0	NA	0	NA	61 ± 1	NA	100 ± 0	NA	1	1	NA	NA
CCRG1-L-014	14 ± 1	NA	100 ± 0	NA	100 ± 0	NA	100 ± 0	NA	91 ± 1	NA	52 ± 1	NA	37 ± 2	NA	100 ± 0	NA	1	1	NA	NA
CCRG1-L-017	11 ± 1	NA	100 ± 0	NA	100 ± 0	NA	100 ± 0	NA	100 ± 0	NA	65 ± 1	NA	1 ± 0.5	NA	100 ± 0	NA	1	0	NA	NA
CCRG1-L-023	15 ± 1	3 ± 3	100 ± 0	96 ± 6	100 ± 0	100 ± 0	100 ± 0	100 ± 0	100 ± 0	70 ± 4	0	0	10 ± 1	1 ± 2	100 ± 0	100 ± 0	1	0	0.97	0.0001
CCRG1-L-024	10 ± 1	1 ± 1	100 ± 0	53 ± 8	100 ± 0	20 ± 18	100 ± 0	87 ± 13	100 ± 0	72 ± 16	0	0	4 ± 1	22 ± 11	100 ± 0	1 ± 1	1	0	0.58	0.12
CCRG1-L-026	6 ± 1	0	100 ± 0	87 ± 5	100 ± 0	57 ± 16	100 ± 0	85 ± 6	100 ± 0	65 ± 12	30 ± 1	0	9 ± 1	25 ± 17	100 ± 0	100 ± 0	0	0	0.9	0.002
CCRG1-L-065	12 ± 1	0	100 ± 0	95 ± 5	100 ± 0	46 ± 28	100 ± 0	0	100 ± 0	88 ± 13	9 ± 1	2 ± 2	1 ± 1	64 ± 8	100 ± 0	100 ± 0	0	0	0.49	0.22
CCRG1-L-066	17 ± 2	3 ± 1	100 ± 0	100 ± 0	100 ± 0	88 ± 11	100 ± 0	73 ± 18	100 ± 0	93 ± 8	2 ± 1	15 ± 7	10 ± 1	9 ± 10	100 ± 0	100 ± 0	1	0	0.93	0.0001
CCRG1-L-070	18 ± 1	55 ± 4	100 ± 0	100 ± 0	100 ± 0	100 ± 0	100 ± 0	100 ± 0	100 ± 0	77 ± 11	64 ± 2	38 ± 16	6 ± 1	72 ± 13	100 ± 0	79 ± 6	1	0	0.6	0.11
CCRG1-L-075	37 ± 1	25 ± 18	100 ± 0	100 ± 0	100 ± 0	100 ± 0	100 ± 0	100 ± 0	100 ± 0	100 ± 0	0	4 ± 4	95 ± 3	58 ± 12	100 ± 0	93 ± 12	1	1	0.88	0.0005
CCRG1-L-087	12 ± 2	20 ± 5	100 ± 0	100 ± 0	100 ± 0	100 ± 0	100 ± 0	100 ± 0	96 ± 1	100 ± 0	44 ± 1	2 ± 2	46 ± 2	71 ± 14	100 ± 0	100 ± 0	1	0	0.9	0.003
CCRG1-L-088	16 ± 1	4 ± 3	100 ± 0	100 ± 0	100 ± 0	27 ± 3	100 ± 0	100 ± 0	84 ± 1	100 ± 0	51 ± 1	80 ± 8	21 ± 1	79 ± 18	100 ± 0	100 ± 0	1	0	0.5	0.21

**Table 5:** Summary of the single base substitution (SBS), small insertion and deletion (ID) and doublet base substitution (DBS) signatures in PDES and cell lines. The SBS, ID, and DBS signatures from the COSMIC database identified after the removal of common variants using dbSNP and gnomAD are shown. SBS5, ID1, ID2, and ID12 were the most common signatures in PDES and ES cell lines. ES cell lines contained additional SBS signatures (SBS7c, SBS8, SBS10c, SBS18, SBS30, and SBS40). The assignment confidence of each signature with individual PDES and cell lines is reported. SBS and ID signatures were assigned with high confidence (>0.71). However, DBS signatures were assigned with low confidence (<0.49) and are therefore excluded. Green = signature observed, red = signature not observed. The total number of SBS, ID, and DBS mutations observed in each PDES and cell line is shown. AC = Assignment Confidence.

Sample ID	Total SBS Mutations	SBS1	SBS7c	SBS5	SBS8	SBS10c	SBS18	SBS30	SBS40	SBS AC	Total ID Mutations	ID1	ID2	ID10	ID12	ID AC	Total DBS Mutations	DBS3	DBS7	DBS9	DBS11	DBS AC
A673	13,260									0.87	48,969					0.75	323					0.29
RDES	12,989									0.89	46,800					0.75	293					0.23
SKES-1	13,182									0.87	48,074					0.76	286					0.26
SK-N-MC	14,991									0.89	48,924					0.74	317					0.49
TC-32	16,651									0.88	48,906					0.71	330					0.35
TTC 466	15,804									0.91	50,787					0.74	323					0.12
CCRG1-L-008	11,643									0.88	48,831					0.72	305					0.19
CCRG1-L-014	12,012									0.85	49,161					0.71	322					0.22
CCRG1-L-017	12,503									0.88	48,388					0.72	250					0.30
CCRG1-L-023	11,543									0.87	47,846					0.74	281					0.18
CCRG1-L-066	11,994									0.85	50,024					0.72	304					0.22
CCRG1-L-087	12,983									0.87	50,278					0.74	321					0.20
CCRG1-L-088	13,776									0.88	54,678					0.87	310					0.10

**Table 6:** Migration of PDES and ES cell lines. The migration index (MI ± SEM) of PDES and ES cell lines was calculated by expressing the total migrated area at 72 h relative to the size of the spheroid core at 0 h. Migration was observed in 100% of PDES (25/25) and ES cell lines (6/6); PDES were significantly more migratory than cell lines (*p* < 0.02).

Cell Culture	Migration Index (MI ± SEM)
A673	26 ± 4
RD-ES	22 ± 3
SKES-1	7 ± 0.4
SK-N-MC	12 ± 1
TC-32	25 ± 2
TTC466	29 ± 4
CCRG1-L-001	97 ± 12
CCRG1-L-003	31 ± 1
CCRG1-L-005	100 ± 2
CCRG1-L-006	56 ± 5
CCRG1-L-008	35 ± 3
CCRG1-L-009	96 ± 2
CCRG1-L-011	149 ± 5
CCRG1-L-014	25 ± 2
CCRG1-L-017	49 ± 5
CCRG1-L-023	11 ± 6
CCRG1-L-024	13 ± 2
CCRG1-L-026	19 ± 2
CCRG1-L-031	62 ± 31
CCRG1-L-032	73 ± 9
CCRG1-L-033	60 ± 5
CCRG1-L-035	68 ± 2
CCRG1-L-036	57 ± 3
CCRG1-L-037	171 ± 4
CCRG1-L-065	36 ± 4
CCRG1-L-066	32 ± 3
CCRG1-L-070	14 ± 1
CCRG1-L-073	45 ± 7
CCRG1-L-075	16 ± 1
CCRG1-L-087	10 ± 1
CCRG1-L-088	69 ± 2

**Table 7:** Differential expression of EWSR1 fusion activated and repressed target genes in PDES and cell lines. RNA levels of downstream targets of the EWSR1-FLI1 [37,63,64,71,72], detected using RNA sequencing and normalised using DESeq2 (Table S2), were significantly increased (10/17 activated targets) and decreased (9/11 repressed targets) in PDES compared to cell lines. The fold-change in expression of each gene is presented as Log_2_ Fold Change, where a positive value is an increase in cell lines compared to PDES and a negative value is a decrease in gene expression in cell lines compared to PDES. The adjusted *p*-value (*p*-value from the Wald test, corrected for multiple testing using the Benjamini–Hochberg method) for each differentially expressed gene is shown.

Gene (Italics = Significant Difference in Expression)	Reported Change in Target Expression by EWSR1 Fusion (I = Increase, D = Decrease, NR = Not Regulated by EWSR1 Fusion)	Log2fold Change in Expression (Positive Value = Increased Expression in Cell Lines Compared to PDES, Negative Value = Decreased Expression in Cell Lines Compared to PDES)	Adjusted *p*-Value	Protein Expression Analysed in PDES and ES Cell Lines by Immunofluorescence (1 = Yes, 0 = No)
CCND1 [64]	I	0.6	0.090	0
E2F3 [64]	I	3.3	0.220	0
NKX2-2 [64,71]	I	2.4	0.300	1
NR0B1 [64,71]	I	3.2	0.080	0
PAX7 [71]	I	3.2	0.073	0
PRKCB [71]	I	3.3	0.110	0
SPRY1 [71]	I	−1.1	0.054	0
FOXO1 [64]	D	0.5	0.420	0
NCAM1 [64]	D	−1.5	0.300	0
*BCL11B* [64]	I	3.7	0.002	0
*CCK* [71]	I	4.6	0.038	0
*FEZF1* [64]	I	0.8	5 × 10^−5^	0
*FOXM1* [71]	I	1.7	4.49 × 10^−6^	0
*GLI1* [64]	I	1.9	6.3 x10^−5^	0
*MEIS1* [64]	I	1.7	2.5 × 10^−10^	0
*MYBL2* [71]	I	2.5	2.91 × 10^−5^	0
*SOX2* [64,71]	I	4.6	0.048	1
*SOX6* [71]	I	1.9	1.05 × 10^−5^	0
*STEAP1* [71]	I	2.0	1.43 × 10^−5^	0
*CD73/NT5E* [63]	D	−4.1	3.7 × 10^−18^	1
*N-cadherin/CDH2* [72]	D	−3.4	4.38 × 10^−8^	0
*HOXD13* [64]	D	3.6	0.010	0
*ITGA1* [64]	D	−1.8	3.5 × 10^−8^	0
*ITGA4* [64]	D	−2.6	2.6 × 10^−9^	0
*ITGB1* [64]	D	−2.4	4.1 × 10^−43^	1
*LOX* [71]	D	−3.8	8.1 × 10^−36^	1
*RUNX3* [64]	D	2.2	0.003	0
*SLUG/SNAI2* [72]	D	−3.8	8.27 × 10^−50^	1
ZEB2 [37]	NR	0.9	0.050	1

**Table 8:** Top enriched pathways represented by genes significantly differentially expressed in PDES compared to cell lines. Differentially expressed genes between PDES and cell lines were identified by comparing normalised RNA sequencing expression data using DESeq2. Gene set enrichment (GSE) analysis of differentially expressed genes (DEGs) comparing PDES and cell lines was performed using clusterProfiler. The top 20 pathways (based on *Q* value) represented by all DEGs in cell lines compared to PDES (Table S3) are shown. Database = database associated with the pathway (GO = gene ontology, KEGG = Kyoto Encyclopaedia of Genes and Genomes, REACT = reactome); Ontology = only applicable for GO terms (BP = biological process, CC = cellular component, MF = molecular function, NA = not applicable); Pathway description = description of the pathway; Set Size= total number of DEGs that are assigned to the pathway; Enrichment score (EScore) = degree to which DEGs are over-represented in the ranked list; Normalised enrichment score (NES) = allows comparability across gene sets by accounting for differences in gene set size; positive EScore/NES = pathway enriched in cell lines; negative EScore/NES = pathway suppressed in cell lines; *p*-value = generated by a one-sided Fisher’s exact test; adjusted *p*-value = hypergeometric *p*-value after correction for multiple testing; *Q* value = FDR adjusted *p*-value; Rank = gene list order based on Log_2_ Fold Change.

Database	Ontology (NA = Not Applicable)	Pathway ID	Pathway Description	Set Size (Total Number of DEGs That Are Assigned to the Pathway)	Enrichment Score (Degree by Which DEGs Are Over-Represented in the Ranked List)	Normalised Enrichment Score (Normalised Enrichment Score by Accounting for Differences in the Gene Set Size)	*p*-Value	Adjusted *p*-Value	*Q* Value	Rank
GO	CC	GO:0062023	Collagen-containing extracellular matrix	226	−0.562465709	−2.695796296	1.00 × 10^−10^	6.66 × 10^−9^	4.78 × 10^−9^	1448
GO	CC	GO:0031012	Extracellular matrix	283	−0.553418777	−2.681809517	1.00 × 10^−10^	6.66 × 10^−9^	4.78 × 10^−9^	1526
GO	CC	GO:0030312	External encapsulating structure	284	−0.550208013	−2.670145215	1.00 × 10^−10^	6.66 × 10^−9^	4.78 × 10^−9^	1526
GO	CC	GO:0098687	Chromosomal region	155	0.408868515	2.641934734	1.00 × 10^−10^	6.66 × 10^−9^	4.78 × 10^−9^	2608
GO	CC	GO:0030016	Myofibril	133	−0.53570449	−2.455482119	1.00 × 10^−10^	6.66 × 10^−9^	4.78 × 10^−9^	1811
GO	CC	GO:0043292	Contractile fiber	138	−0.527960201	−2.428793471	1.00 × 10^−10^	6.66 × 10^−9^	4.78 × 10^−9^	1811
GO	CC	GO:0030017	Sarcomere	118	−0.535275342	−2.40809321	1.00 × 10^−10^	6.66 × 10^−9^	4.78 × 10^−9^	1811
REACT	NA	R-HSA−72203	Processing of Capped Intron-Containing Pre-mRNA	75	0.525853194	3.047154236	1.00 × 10^−10^	1.78 × 10^−8^	1.34 × 10^−8^	2695
REACT	NA	R-HSA−1640170	Cell Cycle	244	0.3968798	2.832919078	1.00 × 10^−10^	1.78 × 10^−8^	1.34 × 10^−8^	2500
REACT	NA	R-HSA−8953854	Metabolism of RNA	154	0.415398577	2.762211119	1.00 × 10^−10^	1.78 × 10^−8^	1.34 × 10^−8^	2695
REACT	NA	R-HSA−69278	Cell Cycle, Mitotic	200	0.385397378	2.717167682	1.00 × 10^−10^	1.78 × 10^−8^	1.34 × 10^−8^	2595
REACT	NA	R-HSA−1474244	Extracellular matrix organisation	185	−0.530974664	−2.498493976	1.00 × 10^−10^	1.78 × 10^−8^	1.34 × 10^−8^	1657
GO	MF	GO:0005201	Extracellular matrix structural constituent	102	−0.587412168	−2.564252047	1.00 × 10^−10^	1.67 × 10^−8^	1.37 × 10^−8^	1204
GO	MF	GO:0005539	Glycosaminoglycan binding	119	−0.553289044	−2.475037826	1.00 × 10^−10^	1.67 × 10^−8^	1.37 × 10^−8^	1477
GO	MF	GO:0008201	Heparin binding	87	−0.577249975	−2.468504872	1.05 × 10^−10^	1.67 × 10^−8^	1.37 × 10^−8^	1269
GO	MF	GO:0005198	Structural molecule activity	296	−0.431948171	−2.076791079	1.00 × 10^−10^	1.67 × 10^−8^	1.37 × 10^−8^	1339
REACT	NA	R-HSA−6805567	Keratinisation	42	−0.700056529	−2.609451378	1.62 × 10^−10^	2.40 × 10^−8^	1.80 × 10^−8^	1163
KEGG	NA	hsa04820	Cytoskeleton in muscle cells	159	−0.530857311	−2.474503248	1.00 × 10^−10^	2.97 × 10^−8^	2.38 × 10^−8^	1683
GO	BP	GO:0006261	DNA-templated DNA replication	80	0.508975338	2.993410783	1.00 × 10^−10^	3.64 × 10^−8^	2.70 × 10^−8^	2199
GO	BP	GO:0045229	External encapsulating structure organisation	183	−0.520582429	−2.442111815	1.00 × 10^−10^	3.64 × 10^−8^	2.70 × 10^−8^	1591

**Table 9:** Clinical information and the EC50 values of chemotherapies, zoledronic acid, mTKIs, and trabectedin in PDES and cell lines. The EC50 values of doxorubicin, etoposide, vincristine, 4-hyperoxyifosfamide (the active metabolite of ifosfamide), zoledronic acid, and the mTKIs cabozantinib, lenvatinib, regorafenib, and trabectedin after treatment for 48 h in 9 PDES and 2 ES cell lines are summarized. Cells were labelled with DAPI and TOTO-3, and the number of cells remaining after treatment was determined by HCI. Cell lines were more sensitive to chemotherapy, ionising radiation, mTKIs, and trabectedin than PDES. There was no association between response to treatment of PDES and corresponding patient tumour necrosis, event-free, or overall survival. NS = no surgery, NK = clinical information not known, NA = not applicable, ND = EC50 not determined, black * = *p* < 0.0001 compared to TC-32, red * = *p* < 0.0001 compared to RD-ES unless stated.

	Original Sample Type (PDES Isolated from Tumour at Diagnosis = 1 or Relapse = 2)	Response to Chemotherapy (Percent of Tumour Necrosis at Resection)	Relapsed Disease (No Relapse = 0, Relapse = 1, Progressive Disease = 2)	Clinical Outcome (Alive = 0, Died = 1) and Number of Days from Diagnosis to Date Last Seen	EC50 Concentration (µM)								
					Doxorubicin	Etoposide	Vincristine	4-Hyperoxyifosfamide	Zoledronic Acid	Cabozantinib	Lenvatinib	Regorafenib	Trabectedin
RD-ES	NA	NA	NA	NA	0.008	0.011	0.012	0.18	5	7.8	24.5	11.9	0.0000001
TC-32	NA	NA	NA	NA	0.005	0.034	0.006	0.04	5.3	0.8	6.2	2.6	0.00000001
CCRG1-L-005	1	NK	1	0 (7397 days)	ND **	ND **	ND **	13.4 **	ND	2.8 **	45.4 ** (*p* = 0.03)	ND **	NA
CCRG1-L-008	1	65%	1	1 (1142 days)	ND **	ND **	ND **	30.7 **	ND	ND **	ND **	ND **	0.004 **
CCRG1-L-014	1	NS	1	1 (702 days)	ND **	ND **	ND **	105.1 **	ND	ND **	ND **	ND **	0.003 **
CCRG1-L-017	1	NS	2	1 (417 days)	ND **	ND **	ND **	13.7 **	ND	10 **	26.1 ** (*p* = 0.02)	ND **	0.001 **
CCRG1-L-023	1	100%	1	1 (1009 days)	ND **	ND **	1.1 **	48.7 **	ND	12.2 **	27.9 ** (*p* = 0.002)	ND **	0.002 **
CCRG1-L-066	1	NS	0	0 (1971 days)	ND **	ND **	0.1 **	34.1 **	ND	9 ** (*p* = 0.02)	6.4 **	29.8 **	0.005 **
CCRG1-L-075	2	NK	NK	NK	0.02 **	0.37 **	0.008 * (*p* = 0.0002) * (*p* = 0.01)	2.3 **	ND	2.2 **	11.3 * (*p* = 0.001) *	6.8 * (*p* = 0.03) *	0.0000005 ** (*p* = 0.03)
CCRG1-L-087	2	100%	1	0 (3817 days)	ND **	ND **	ND **	26.7 **	ND	11.9 **	33.6 ** (*p* = 0.01)	ND **	0.001 **
CCRG1-L-088	2	80–90%	1	0 (2720 days)	ND **	ND **	ND **	11.2 **	ND	ND **	ND ** (*p* = 0.02)	20 **	0.001 **

## Data Availability

The WGS data presented in this study are available on request from the corresponding author to protect anonymisation. The FASTQ files of the RNA sequencing data of PDES are available in the Research Data Leeds Repository (University of Leeds), Burchill, Susan and Roundhill, Elizabeth (2020): Total RNA sequencing of patient-derived Ewing sarcoma and Ewing sarcoma CSCs, University of Leeds. [Dataset] https://doi.org/10.5518/887. Burchill, SA and Roundhill, EA (2023) Total RNA sequencing of Ewing sarcoma cell lines and 3D spheroids. University of Leeds. [Dataset] https://doi.org/10.5518/1210.

## Supplementary Materials

The following supporting information can be downloaded at: https://www.mdpi.com/article/10.3390/cancers18030512/s1, Figure S1: Full images of Western blots for EWSR1-FLI1 (68 kDa) using antibodies to the FLI1 protein. EWSR1-ERG (68 kDa) was not detected in the PDES studied; Western blot using antibodies to ERG. Equal protein loading was confirmed following incubation with an antibody to β-actin. Protein extracts from TC-32 (EWSR1-FLI type 1), RD-ES (EWSR1-FLI type 2) and TTC 466 (EWSR1-ERG) cells were included as positive controls; SH-SY-5Y neuroblastoma cell protein extract was included as a negative control. MW = molecular weight markers. Figure S2: The correlation in target protein expression between each PDES and the corresponding tumour tissue from which it was derived; Figure S3: The expression level of EWSR1 fusion activated and repressed targets in PDES and cell lines; Figure S4: The response of PDES and ES cell lines to ionising radiation at 48 h, Table S1: Passage of PDES cells in experiments. Table S2: Differentially expressed genes (DEGs) comparing 6 ES cell lines and 26 PDES; Table S3: GO, KEGG and reactome pathways represented by all differentially expressed genes (DEGs) in cell lines compared to PDES; Table S4: The normalised read counts for reported targets of mTKIs in PDES and cell lines.
