# Supplementary material for: Characterisation of Bespoke Patient-Derived In Vitro Models of Ewing Sarcoma
Source: Cancers (Basel). 2026 Feb 4;18(3):512. doi: 10.3390/cancers18030512 (PMC12897393; doi:10.3390/cancers18030512)
Supplement: Supplementary file 1 [file cancers-18-00512-s001.zip › cancers-4080986-supplementary.pdf]

Supplementary Figure S1

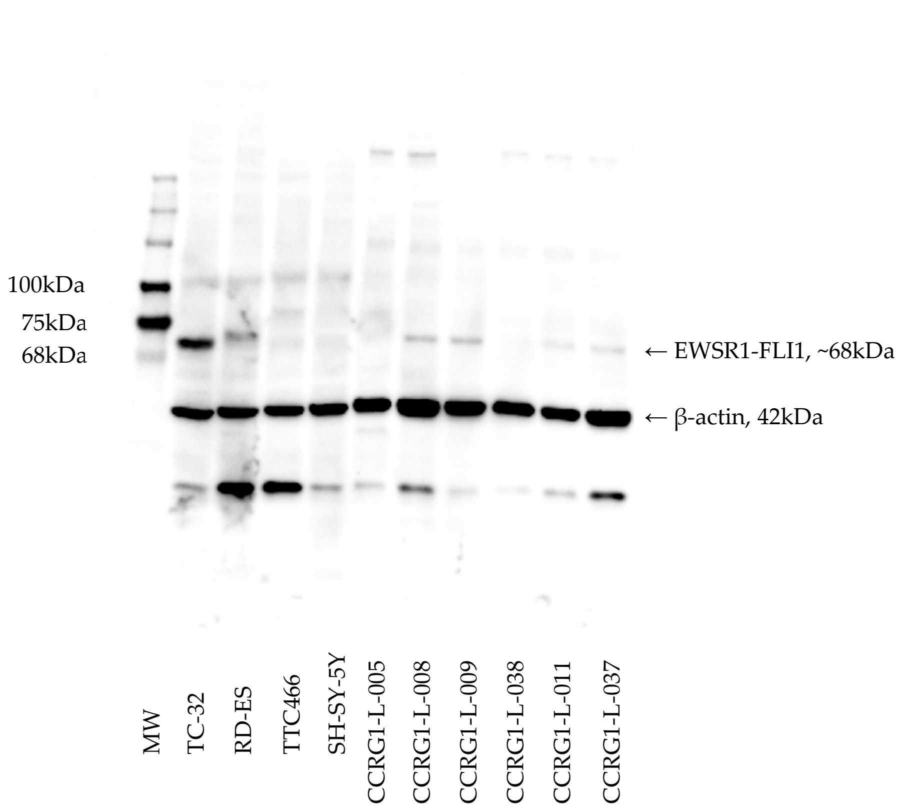


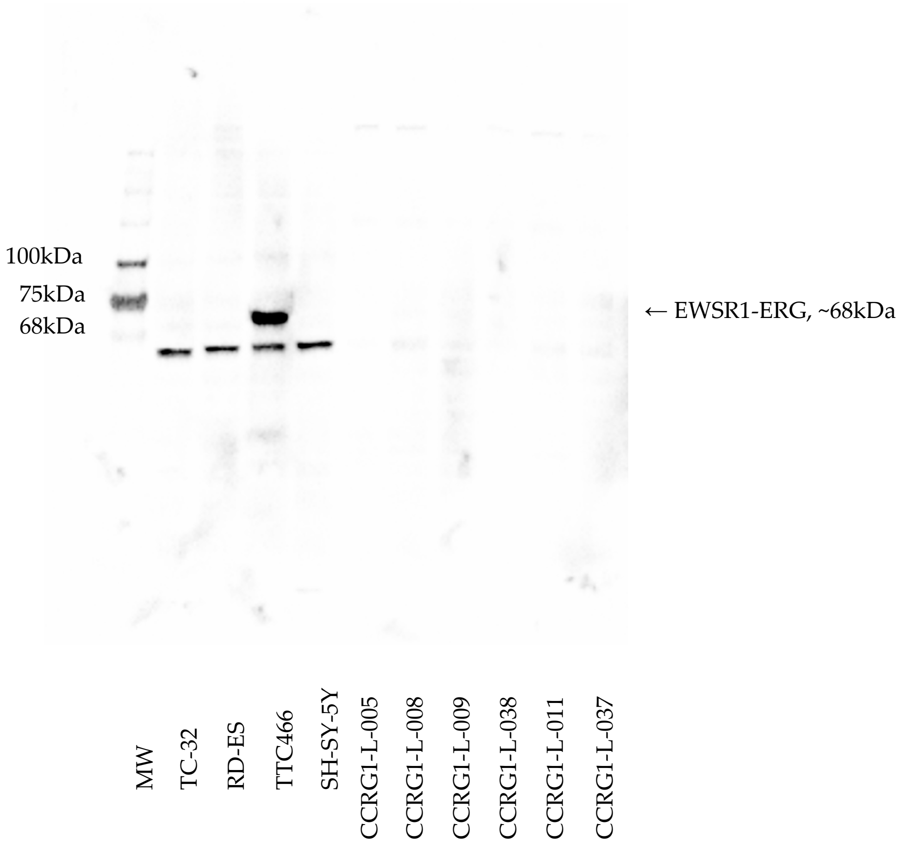

# Supplementary Figure S2

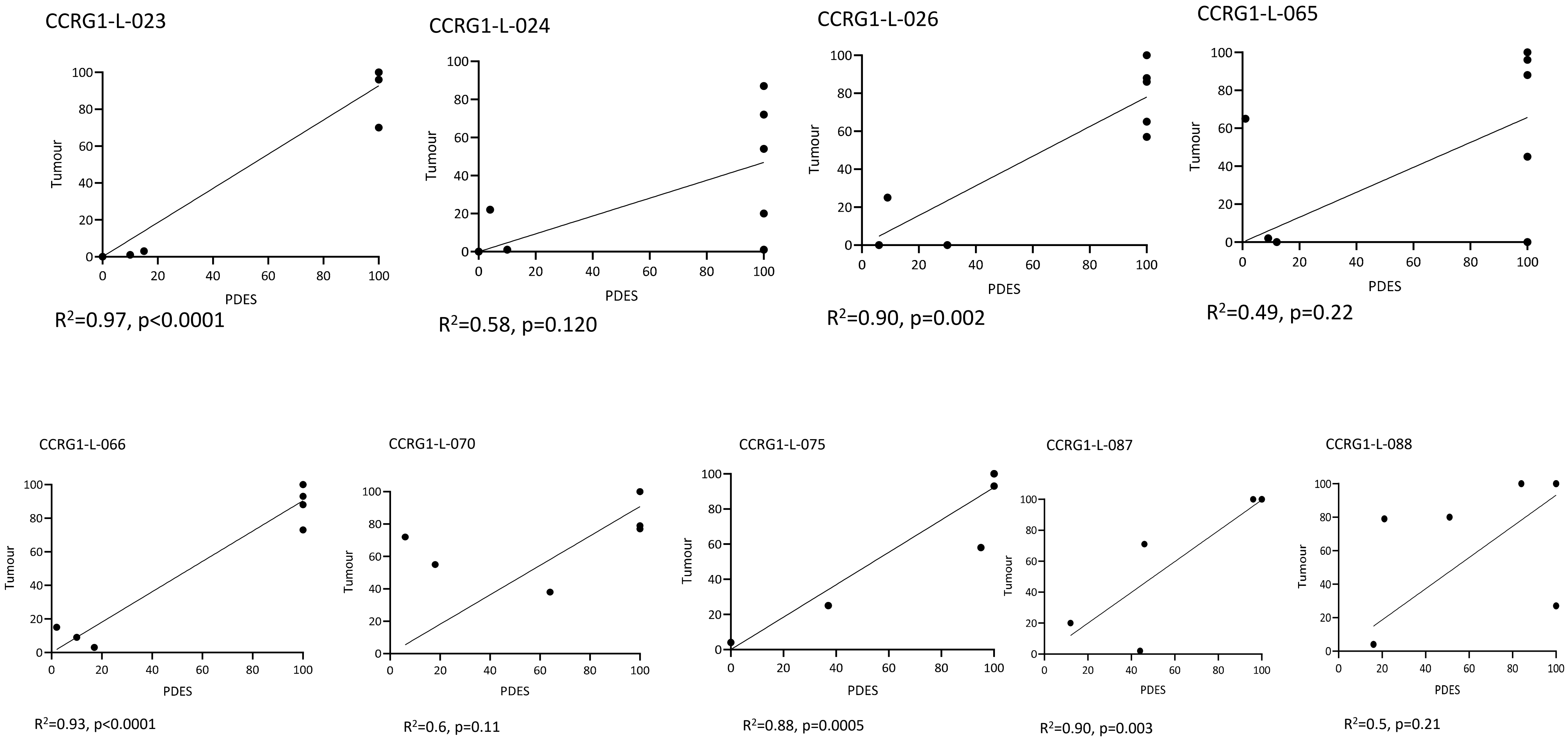

Supplementary Figure S3

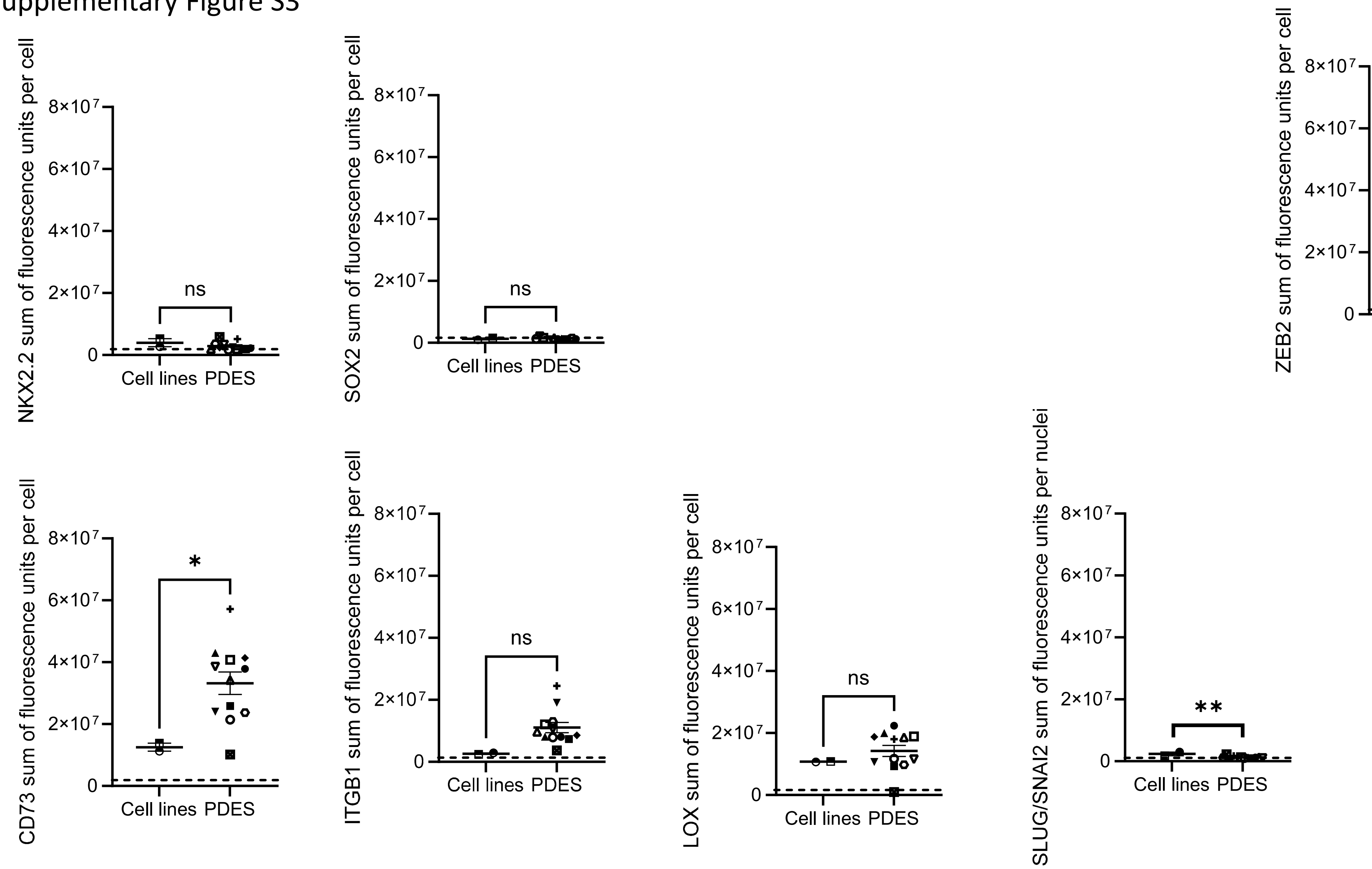


Open circ=5366
Open square 15000
Open triangle=3525
Filled triangle=4050, filled circ=5366, filled square=8144, filled diamond=14465, half circ=tc32, half square=RDES, 9660 open hex, 7894 cross, 7007 upside down triangle open, 5822 filled upside down triangle

Supplementary Figure S4

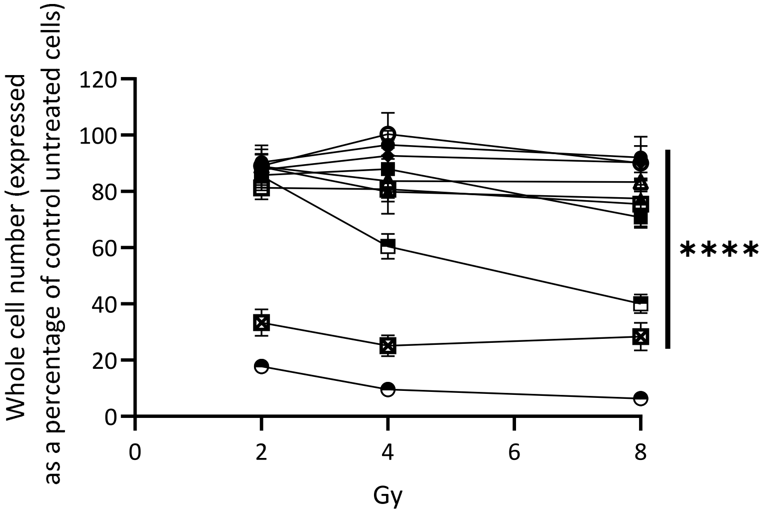

# Supplementary Table S1

| Cell culture | Passage used in manuscript |
|---|---|
| CCRG1-L-005 | p8 to p11 |
| CCRG1-L-008 | p10 to p13 |
| CCRG1-L-014 | p11 to p14 |
| CCRG1-L-017 | p10 to p13 |
| CCRG1-L-023 | p12 to p15 |
| CCRG1-L-024 | p3 and p6 |
| CCRG1-L-026 | p4 and p7 |
| CCRG1-L-065 | p4 and p6 |
| CCRG1-L-066 | p12 to p16 |
| CCRG1-L-070 | p4 and p8 |
| CCRG1-L-075 | p8 to p14 |
| CCRG1-L-087 | p9 and p12 |
| CCRG1-L-088 | p9 and p13 |

Supplementary Table S2

See excel file: Supplementary Table S2.xlsx

Supplementary Table S3

See excel file: Supplementary Table S3.xlsx

Supplementary Table S4

| Gene | Target of TKI | CCRG1-L-005 | CCRG1-L-008 | CCRG1-L-014 | CCRG1-L-017 | CCRG1-L-023 | CCRG1-L-066 | CCRG1-L-087 | RD-ES | TC-32 |
|---|---|---|---|---|---|---|---|---|---|---|
| **AXL** | **Cabozantinib** | 23879 | 38196 | 60604 | 102128 | 38717 | 10314 | 8815 | 110 | 67 |
| **FGFR3** | **Cabozantinib** | 13 | 80 | 244 | 147 | 115 | 34 | 21 | 152 | 226 |
| **FGFR4** | **Cabozantinib** | 4 | 2452 | 2361 | 44 | 673 | 3 | 3 | 567 | 99 |
| **FLT-3** | **Cabozantinib** | 0 | 0 | 0 | 0 | 2 | 0 | 0 | 8 | 0 |
| **MET** | **Cabozantinib** | 5692 | 10390 | 11800 | 32116 | 20031 | 4566 | 1132 | 51 | 52 |
| **TRKB** | **Cabozantinib** | 0 | 63 | 24 | 554 | 318 | 18 | 26 | 5 | 1 |
| **Abl** | **Regorafenib** | 7668 | 15759 | 31872 | 21277 | 11795 | 2140 | 1380 | 2561 | 2919 |
| **BRAF** | **Regorafenib** | 2330 | 2780 | 5817 | 4504 | 2339 | 765 | 548 | 1443 | 1360 |
| **DDR2** | **Regorafenib** | 32149 | 30232 | 77648 | 77032 | 46599 | 8014 | 4442 | 3253 | 29924 |
| **Eph2A** | **Regorafenib** | 1649 | 3406 | 5367 | 7655 | 4514 | 1238 | 429 | 2387 | 687 |
| **PDGFRβ** | **Regorafenib** | 18487 | 17493 | 53638 | 19182 | 16422 | 2500 | 4633 | 531 | 391 |
| **PTK5** | **Regorafenib** | 57 | 225 | 510 | 86 | 70 | 25 | 24 | 47 | 209 |
| **RAF1** | **Regorafenib** | 5539 | 6742 | 15782 | 17819 | 8076 | 993 | 667 | 3305 | 3047 |
| **SAPK2** | **Regorafenib** | 869 | 1108 | 2786 | 2301 | 1547 | 249 | 93 | 398 | 212 |
| **TRKA** | **Regorafenib** | 9 | 26 | 25 | 5 | 17 | 4 | 0 | 48 | 98 |
| **FGFR1** | **Lenvatinib, regorafenib** | 5395 | 9109 | 18320 | 16832 | 11396 | 3042 | 1024 | 3757 | 2405 |
| **FGFR2** | **Lenvatinib, regorafenib** | 57 | 1224 | 2785 | 425 | 728 | 320 | 2 | 112 | 2 |
| **PDGFRα** | **Lenvatinib, regorafenib** | 7385 | 17534 | 15126 | 25773 | 27153 | 5941 | 2089 | 21 | 5 |
| **TIE2** | **Regorafinib, cabozantinib** | 6368 | 2695 | 13551 | 704 | 1786 | 130 | 589 | 7 | 0 |
| **KIT** | **Lenvatinib, regorafenib, cabozantinib** | 32 | 189 | 608 | 898 | 233 | 89 | 32 | 3506 | 5580 |
| **RET** | **Lenvatinib, regorafenib, cabozantinib** | 5 | 22 | 28 | 24 | 13 | 1 | 2 | 633 | 770 |
| **VEGFR1** | **Lenvatinib, regorafenib, cabozantinib** | 4 | 53 | 129 | 452 | 121 | 3 | 107 | 3 | 5 |
| **VEGFR2** | **Lenvatinib, regorafenib, cabozantinib** | 0 | 7 | 7 | 20 | 0 | 1 | 0 | 6 | 0 |
| **VEGFR3** | **Lenvatinib, regorafenib, cabozantinib** | 1 | 24 | 52 | 14 | 13 | 0 | 1 | 347 | 33 |
